# The chemotaxonomic classification of *Rhodiola* plants and its correlation with morphological characteristics and genetic taxonomy

**DOI:** 10.1186/1752-153X-7-118

**Published:** 2013-07-12

**Authors:** Zhenli Liu, Yuanyan Liu, Chunsheng Liu, Zhiqian Song, Qing Li, Qinglin Zha, Cheng Lu, Chun Wang, Zhangchi Ning, Yuxin Zhang, Cheng Tian, Aiping Lu

**Affiliations:** 1 Institution of Basic Theory, China Academy of Chinese Medical Sciences, Beijing, China; 2 School of Chinese Materia Medica, Beijing Municipal Key Laboratory for Basic Research of Chinese Medicine, Beijing University of Chinese Medicine, Beijing, China; 3 Institute of Basic Research in Clinical Medicine, China Academy of Chinese Medical Sciences, Beijing 100700, China; 4 School of Chinese Medicine, Hong Kong Baptist University, Kowloon Tong, Hong Kong SAR, China

**Keywords:** *Rhodiola* plants, Morphological characteristic, Genetic taxonomy, Phytochemical taxonomy

## Abstract

**Background:**

*Rhodiola* plants are used as a natural remedy in the western world and as a traditional herbal medicine in China, and are valued for their ability to enhance human resistance to stress or fatigue and to promote longevity. Due to the morphological similarities among different species, the identification of the genus remains somewhat controversial, which may affect their safety and effectiveness in clinical use.

**Results:**

In this paper, 47 *Rhodiola* samples of seven species were collected from thirteen local provinces of China. They were identified by their morphological characteristics and genetic and phytochemical taxonomies. Eight bioactive chemotaxonomic markers from four chemical classes (phenylpropanoids, phenylethanol derivatives, flavonoids and phenolic acids) were determined to evaluate and distinguish the chemotaxonomy of *Rhodiola* samples using an HPLC-DAD/UV method. Hierarchical cluster analysis (HCA) and principal component analysis (PCA) were applied to compare the two classification methods between genetic and phytochemical taxonomy.

**Conclusions:**

The established chemotaxonomic classification could be effectively used for *Rhodiola* species identification.

## Background

The genus *Rhodiola* L. (Crassulaceae) comprises approximately 96 species found in the alpine regions of Asia and Europe. A total of 73 species, 2 subspecies and 7 varieties are found in China [1,2]. *Rhodiola* species, historically used as adaptogens in Russia and northern Europe and as a traditional herbal medicine in China, are valued for their ability to enhance human resistance to stress or fatigue and to promote longevity [3-5]. *Rhodiola* plants are mainly distributed in southwest and northwest of China, with most species located in Tibet and in Sichuan province. In China, the *Rhodiola* species called Hongjingtian have been used as an important adaptogen, hemostatic, and tonic in traditional Tibetan medicines for thousands of years [6]. The phytochemical extracts of *Rhodiola* plants are widely used throughout Europe, Asia and the United States, with biological activities including anti-allergenic and anti-inflammatory effects and enhanced mental alertness, as well as a variety of other therapeutic applications [5]. Because of their commercial utility, *Rhodiola* plants are now cultivated in many locations in Europe and Asia. Most notably, the roots and rhizomes of *R. crenulata* (*RC*) have high activities and have been accepted by the Pharmacopoeia of China [7]. In addition, many *Rhodiola* plants, such as *R. sachalinensis* (*RS*), *R. himalensis* (D. Dons) S. H. Fu (*RH*)*, R. serrata* H. Ohba (*RSE*), *R. rosea L.* (*RR*)*, R. kirilowii (Regel)* Maxim (*RK*) and *R. fastigiata* (HK. F. et Thoma) S. H. Fu (*RF*), etc., are also used as Hongjingtian in China. However, the identification of the closely related species of *Rhodiola* plants is often difficult due to their generally similar morphology.

Phytochemical investigations show that there are six important classes of constituents in *Rhodiola* rhizomes, including phenylpropanoids, phenylethanol derivatives, flavonoids, monoterpernes, triterpenes and phenolic acids [8-10]. Using animal models, bioassay-guided fractionation of various extracts of plant adaptogens have shown that the active components are mainly phenylpropanoids and phenylethanol derivatives, including salidroside, rosavin and tyrosol [4,11-15]. In the Chinese Pharmacopoeia, salidroside is chosen as a marker compound for quality control [7]. Phenylpropanoids, such as rosarin, rosavin and rosin, are not only typical for *Rhodiola* rhizomes but are also pharmacologically active as antioxidants and neuro-stimulants [16-18]. Compounds such as tyrosol and gallic acid had been proven to be good radical scavengers [19,20]. A recent study revealed that rhodionin and salidroside might have anti-tumor effects [21,22]. Additionally, rhodionin is recognized to be involved in learning and memory [23,24]. The above 8 compounds from four chemical classes were selected as chemotaxonomic markers in the present paper. Because they exhibit variety bioactivities, meanwhile, phenylpropanoids and phenylethanol derivatives are characteristic in *Rhodiola* plants. Therefore, it is worthwhile to study the variety of co-existing phytochemical constituents in the plant, which may be responsible for its unique pharmacological activity.

The current taxonomical status of the genus *Rhodiola* has become quite complex. The rationale and defining criteria for the boundaries of the genus remain somewhat controversial [25]. The morphologies of different species of commercial *Rhodiola* plants are too similar to distinguish visually. With the development of DNA sequencing methods and the discovery of the polymerase chain reaction (PCR) for DNA amplification, biological systematic analysis has increasingly been based on DNA sequence analysis. The genotypes identified by PCR amplification suffice to predict the species of plants. In addition to genetic taxonomy and other classical morphological and non-morphological methods, phytochemical taxonomy can also provide supplementary information in species identification [26]. The chemotype of a plant species has traditionally been defined as by profile of natural products, and the genotype has been defined as its genetic constitution or DNA sequence.

Here, we collected 47 *Rhodiola* samples used clinically in different provinces of China. They were identified through morphological characterization and genotyping. In addition, eight bioactive compounds from four chemical classes (phenylpropanoids, phenylethanol derivatives, flavonoids and phenolic acids) were used as chemotaxonomic markers to elucidate the phytochemical taxonomy by an HPLC-DAD/UV method.

## Experimental

### Chemicals and materials

**Figure 1:**
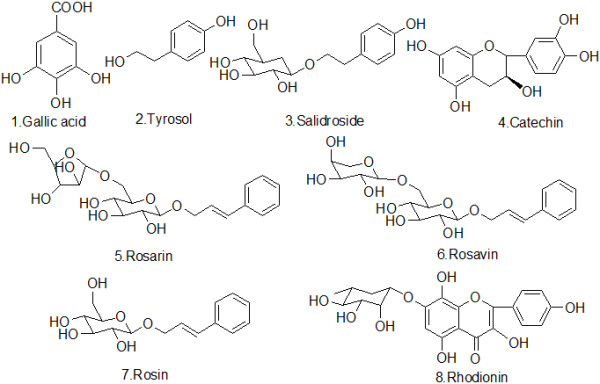
**Chemical structures of the eight reference marker compounds.** Among them, rosarin, rosavin and rosin belong to phenylpropanoids, tyrosol and salidroside belong to phenylethanol derivatives, (+) catechin and rhodionin belong to flavonoids and gallic acid belongs to phenolic acids.

Methanol and acetonitrile were purchased from Fisher Scientific (Pittsburgh, PA, USA). The other reagents were from Beijing Chemical Inc. (Beijing, China). Gallic acid, salidroside and (+) catechin standards were purchased from the National Institutes for Food and Drug Control (Beijing, China). Tyrosol, rhodionin, rosavin, rosarin and rosin standards were obtained from Chromadex (Irvine, CA, USA). The chemical structures of the eight reference marker compounds are listed in Figure 1.

The 47 authentic *Rhodiola* samples were collected from thirteen local provinces of China: Gansu, Jiangxi, Qinghai, Zhejiang, Heilongjiang, Henan, Hebei, Sichuan, Beijing, Neimeng, Liaoning, Xizang and Xinjiang. They were identified as genuine samples of *R. crenulata* (*RC*), *R. sachalinensis* (*RS*), *R. himalensis* (D. Dons) S. H. Fu (*RH*)*, R. serrata* H. Ohba (*RSE*), *R. rosea L.* (*RR*)*, R. kirilowii* (Regel) Maxim (*RK*) *and R. fastigiata* (HK. F. et Thoma) S. H. Fu (*RF*) by Professor Chunsheng Liu. Dried voucher specimens (marked as *RC*-1~*RC*-19, *RS*-20~*RS*-21, *RH*-22, *RSE*-23~*RSE*-37, *RF*-38, *RR*-39 and *RK-*40~*RK*-47) were deposited at the Institute of Basic Theory, China Academy of Chinese Medical Sciences, Beijing, P. R. China, as shown in Table 1.

**Table 1:** **The origins of the 47 *Rhodiola *samples**

**Name**	**NO.**	**Location**	**Name**	**NO.**	**Location**	**Name**	**NO.**	**Location**
RC 1	H7	Gansu	RC 17	H24	Beijing	RSE 33	H17	Xizang
RC 2	H2	Jiangxi	RC 18	H23	Beijing	RSE 34	H25	Xizang
RC 3	H5	Jiangxi	RC 19	H32	Beijing	RSE 35	H31	Hebei
RC 4	H9	Gansu	RS20	-	Liaoning	RSE 36	H28	Beijing
RC 5	H4	Jiangxi	RS21	-	Heilongjiang	RSE 37	H27	Beijing
RC 6	H6	Gansu	RH22	H13	Henan	RF38	H26	Beijing
RC 7	H3	Jiangxi	RSE23	H10	Neimeng	RR39	-	Xinjiang
RC 8	H1	Jiangxi	RSE 24	H11	Neimen	RK 40	-	Qinghai
RC 9	-	Qinghai	RSE 25	H14	Liaoning	RK 41	-	Qinghai
RC 10	-	Zhejiang	RSE 26	H8	Gansu	RK 42	-	Qinghai
RC 11	-	Heilongjiang	RSE 27	H18	Xizang	RK 43	H34	Qinghai
RC 12	-	Henan	RSE 28	H19	Xizang	RK 44	H33	Qinghai
RC 13	-	Gansu	RSE 29	H20	Xizang	RK 45	H35	Qinghai
RC 14	-	Hebei	RSE 30	H21	Xizang	RK 46	-	Qinghai
RC 15	H29	Sichuan	RSE 31	H22	Xizang	RK 47	-	Qinghai
RC 16	H30	Hebei	RSE 32	H16	Xizang			

### HPLC-DAD/UV analysis

The data were obtained using an Agilent 1100 Series HPLC with DAD. The analytical conditions for recording chromatograms of the marker compounds in *Rhodiola* samples were as follows. A Zobax SB-C_18_ column (4.6 mm× 150 mm, 5 μm; Agilent Technologies) was used. The mobile phase consisted of MeCN (A) and 0.2% HAc (B) with a linear gradient elution at a flow rate of 1.0 mL/min. The gradient program (A/B, v/v) was as follows: 5:95 (t = 0 min), 8:92 (t = 10 min), 18:82 (t = 43 min) and 37:63 (t = 60 min). The detection wavelength program was 275 nm (t = 0 min), 250 nm (t = 30 min) and 332 nm (t = 45 min). The column temperature was set to 40°C. The detection wavelength was selected by DAD according to max UV absorption of each reference.

### Sample preparation

The samples were pulverized, and the powder (1.0 g) was accurately weighed and extracted with 25 mL of methanol by ultrasonication for 30 min. After cooling, the solution was filtered through a 0.2 μm membrane filter and stored at 4°C until analysis. A 5-μL aliquot solution was injected for HPLC analysis. Each sample was prepared in triplicate and relative standard deviation (RSD) was calculated for all the samples.

### Method validation

The eight standards were prepared and serially diluted with methanol to obtain seven different concentrations used for plotting standard curves, respectively. Method precision was determined by injecting one Hongjingtian sample solution six times consecutively. Reproducibility was studied through six independently prepared samples from a single batch of Hongjingtian. The stability test was performed by successively injecting the same sample solution over 24 hours. The limit of detection (LOD) and limit of quantity (LOQ) were determined at a signal-to-noise ratio (S/N) of 3 and 10, respectively. Standard solutions were diluted to series of appropriate concentrations with methanol and 5μL aliquots of the diluted solutions were injected into the HPLC for analysis.

### DNA extraction

Nucleic acids were extracted and purified from deep-frozen plant materials. Sample vouchers were deposited in the collection at Beijing University of Chinese Medicine. Genomic DNA was extracted from silica gel-dried root material using a plant DNA extraction kit (Tiangen, Beijing, China) according to the manufacturer’s protocol with some modifications. The quality of the isolated DNA was verified from absorbance measurements at wavelengths 230, 260 and 280 nm and on a 1% (w/v) ethidium bromide-stained agarose gel.

### PCR, cloning and sequencing

The PCR procedure was designed according to the instruction manual of a GeneRacer Kit (Invitrogen, Carlsbad, CA, USA). To achieve the 5′-end cDNA sequence, two rounds of thermal asymmetric interlaced PCR were performed as described in Liu and Chen et al. [27]. All of the obtained fragments were sequenced in both directions by ligating into pGEM&nonBR;T vector and using an ABI 3730XL Genetic Analyzer (Applied Biosystems). PCR was performed using a touchdown strategy: 94°C for 4 min, followed by 10 cycles of 94°C for 75s, 53°C for 5 min, 0.2°C/s to 41°C, and 72°C for 5 min, followed by 35cycles of 94°C for 1 min, 45°C for 2 min, and 72°C for 5 min. The PCR products were run on a 1% (w/v) ethidium bromide-stained agarose gel with a 6 × orange loading buffer (Fermentas, Vilnius, Lithuania). The expected size band (680 bp) was excised from the gel and eluted using a Qiaquick Gel Extraction kit from Qiagen. The eluted PCR product was cloned into pGEM-T Easy Vector (Promega Corporation, Madison, USA) and sequenced using the BigDye Terminator Cycle Sequencing Kit (PE Applied Biosystems, Warrington, UK).The full-length deduced amino acid sequence was aligned with the publicly available HQT groups using ClustalX and MEGA version 4.0 software. A neighbor-joining (NJ) tree was constructed based on standard parameters with bootstrap testing of 1000 replicates. All the DNA sequences obtained were submitted to GenBank-NCBI for comparison with the deposited sequences using the tool BLAST [28].

### Statistical and multivariate analysis

The statistical analysis was performed using the SAS 9.1.3 statistical package (order no. 195557) for PCA and HCA. PCA and HCA were used to show the unsupervised clustering pattern of the *Rhodiola* species. PCA and HCA were used to observe the natural interrelationships among the chemical components for each of the *Rhodiola* samples. The critical *p* value for all analyses in this study was set to 0.05.

## Results and discussion

### Morphological characteristic of the collected samples

The 47 collected *Rhodiola* samples were identified according to their morphological characteristics, and their collection locations are listed in Table 1. According to Flora of China [2], the morphological characteristic of *Rhodiola* plants is as following: stems dimorphic with usually very stout caudex or rhizome, usually with brown or blackish, membranous, scalelike leaves, sharply differentiated from much more slender, erect or ascending, leafy flowering stems. The roots and rhizomes of *Rhodiola* are used as the medicinal parts of the plants. The supplementary characteristic information from the aerial parts of the collected samples could not be obtained in this experiment. The morphologies of some Rhodiola samples are too similar to distinguish visually. In addition, the current taxonomical status of the genus *Rhodiola* has become quite complex. Accordingly, the species of some samples were tentatively identified. Among the 47 *Rhodiola* samples, *RC*-1 ~ *RC*-19 were identified as *R. crenulata*; *RS*-20 ~ *RS*-21 were identified as *R. sachalinensis*; *RH*-22 was identified as *R. himalensis* (D. Dons) S. H. Fu; *RSE*-23 ~ *RSE*-37 were identified as *R. serrata* H. Ohba; *RF*-38 was identified as *R. fastigiata* (HK. F. et Thoma) S. H. Fu; *RR*-39 was identified as *R. rosea* L.; *RK*-40 ~ *RK*-47 were identified as *R. kirilowii* (Regel) Maxim, as shown in Table 1.

### Genetic taxonomic of the collected samples

To verify the accuracy of the identity of the species of the 47 *Rhodiola* samples, a phylogenetic tree was constructed based on the DNA sequences obtained from GenBank. For some samples, it was difficult to extract the exact DNA when the roots had been stored too long and were very dry, even when the roots were contaminated by microbes or the DNA was degraded. Therefore, only 34 *Rhodiola* samples were identified according to their DNA sequences. Among them, 23 *Rhodiola* samples were unambiguously identified with the similarities higher than 98%. The other 11 *Rhodiola* samples were tentatively identified. All sequences were submitted to GenBank (http://www.ncbi.nlm.nih.gov/genbank/) and their IDs were shown in the Additional file 1.

**Figure 2:**
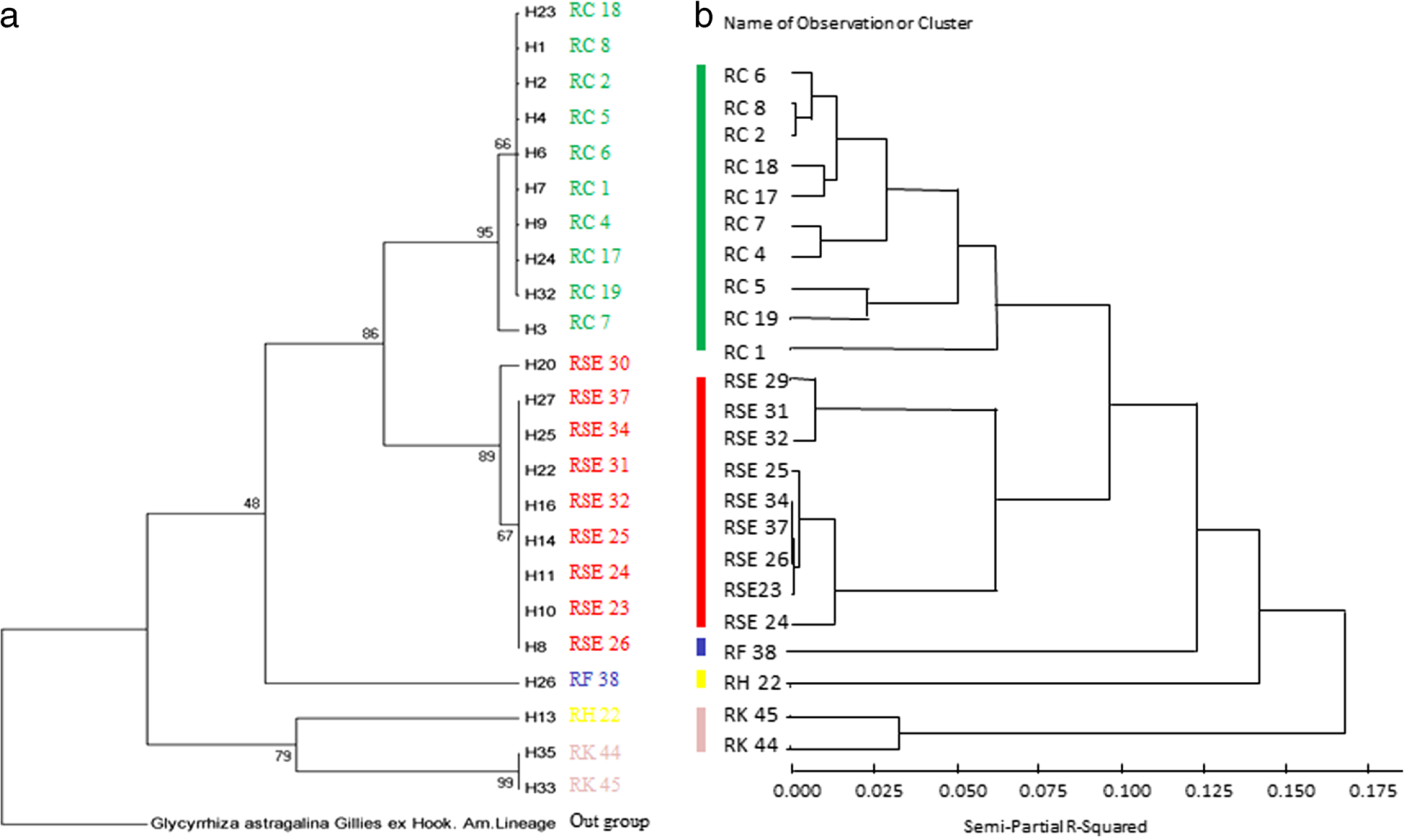
**Hierarchical clustering analysis (HCA) dendrogram according to genetic taxonomy (a) and phytochemical taxonomy (b) derived from 23 *Rhodiola *samples.**

HCA was employed to analyze the genetic data to characterize the population genetics of the *Rhodiola* samples from different geographic regions and determine their genetic diversity and population differentiation (Figure 2a). *Glycyrrhiza astragalina* was selected as an out-group to obtain more precise branching of the phylogenetic tree. The *Rhodiola* populations sampled from China were grouped separately according to their genotype. To avoid subjective errors by operator, the samples were relabeled as H1 ~ H35. The NJ tree obtained from the 23 *Rhodiola* samples was divided into five main characteristic branches. The first branch consisted of *RC*. The second branch included *RSE*. The first branch (*RC*) progressively merged with the second branch (*RSE*) to form a larger cluster that merged with the third branch (*RF*). The fourth branch (*RH*) and the fifth branch (*RK*) together with the above three branches created the whole tree structure.

### Phytochemical taxonomy of the collected samples

Based on the morphological characteristic and genetic taxonomy, both of them have some limitations in species identification. The classification of plants based on chemotypes can be used as a powerful chemotaxonomic tool that provides a detailed view of the differences and similarities between species. The 8 pure bioactive compounds classified into four types were used as chemotaxonomic markers to distinguish among the different *Rhodiola* samples.

#### *Method validation*

To gain high sensitivity and good peak capacity, the chromatographic conditions were optimized, as described in the HPLC-DAD/UV analysis section. MeCN and 0.2%HAc were used as the mobile phase to improve the retention behavior of the constituents on the HPLC column. The wavelength for the detection of compounds was selected by DAD. The chromatograms at 275 nm could provide maximum absorption of gallic acid, tyrosol, salidroside and (+) catechin. The wave length for the detection of rosarin, rosavin and rosin was at 250 nm, and 332 nm was used for the detection of rhodionin.

The method was validated in terms of linearity, LOD and LOQ, precision, reproducibility, stability and recovery test, the results of which are shown in Table 2. Calibration curves were prepared by plotting the peak areas of the marker compounds versus the corresponding concentrations. Good linear relationships (*R*^*2*^ = 1 for rhodionin and 0.9999 for tyrosol, salidroside, rosarin, rosavin, rosin, (+) catechin and gallic acid) were demonstrated over a range of 0.071-737 μg/mL. The accuracy of the analytical method was evaluated using a recovery test. The mean recoveries were from 97.5 to 101.7% with RSD less than 3.0% for the eight reference compounds. The precision of the assay was determined by its reproducibility. The RSD of peak area ranged from 0.49 to 2.47%.

**Table 2:** Linear regression, LODs and LOQs, precisions, reproducibility, stability and recovery for eight compounds

**Compound**	**Regression equation**	***r***	**Linear range**	**LOD**	**LOQ**	**Precision**	**Reproducibility**	**Stability**	**Recover (*n*** **= 6)**	
	**(*n*** **= 2)**		**(μg/mL)**	**(μg/mL)**	**(μg/mL)**	**(*n*** **= 6) RSD (%)**	**(*n*** **= 6) RSD (%)**	**(RSD,%)**	**Recovery (%)**	**RSD (%)**
Salidroside	*Y* = 218.51*X*-0.4	0.9999	3.50-700	0.089	0.27	0.95	1.46	0.53	99.22	2.45
Tyrosol	*Y* = 384.13*X*-3.9	0.9999	0.74-737	0.014	0.043	2.67	2.42	2.40	101.7	1.68
Gallic acid	*Y* = 2923.6*X*-13.8	1	2.52-504	0.042	0.13	0.49	1.80	1.88	98.44	2.85
(+)Catechin	*Y* = 339.77*X* + 3.7	0.9999	3.25-325	0.031	0.15	1.87	2.35	2.11	99.69	2.25
Rosarin	*Y* = 1954.5*X*-2.7	0.9999	3.21-321	0.094	0.36	1.90	2.05	1.67	99.77	2.80
Rosavin	*Y* = 2140.4*X* + 8.4	0.9999	0.071-284	0.0078	0.027	1.97	1.66	1.96	97.51	2.25
Rosin	*Y* = 3076.8*X*-4.2	0.9999	1.25-375	0.037	0.18	1.45	1.19	1.10	100.5	2.77
Rhodionin	*Y* = 166.15*X* + 1.3	0.9999	0.18-183	0.0094	0.032	2.32	2.12	2.61	99.4	1.72

**Figure 3:**
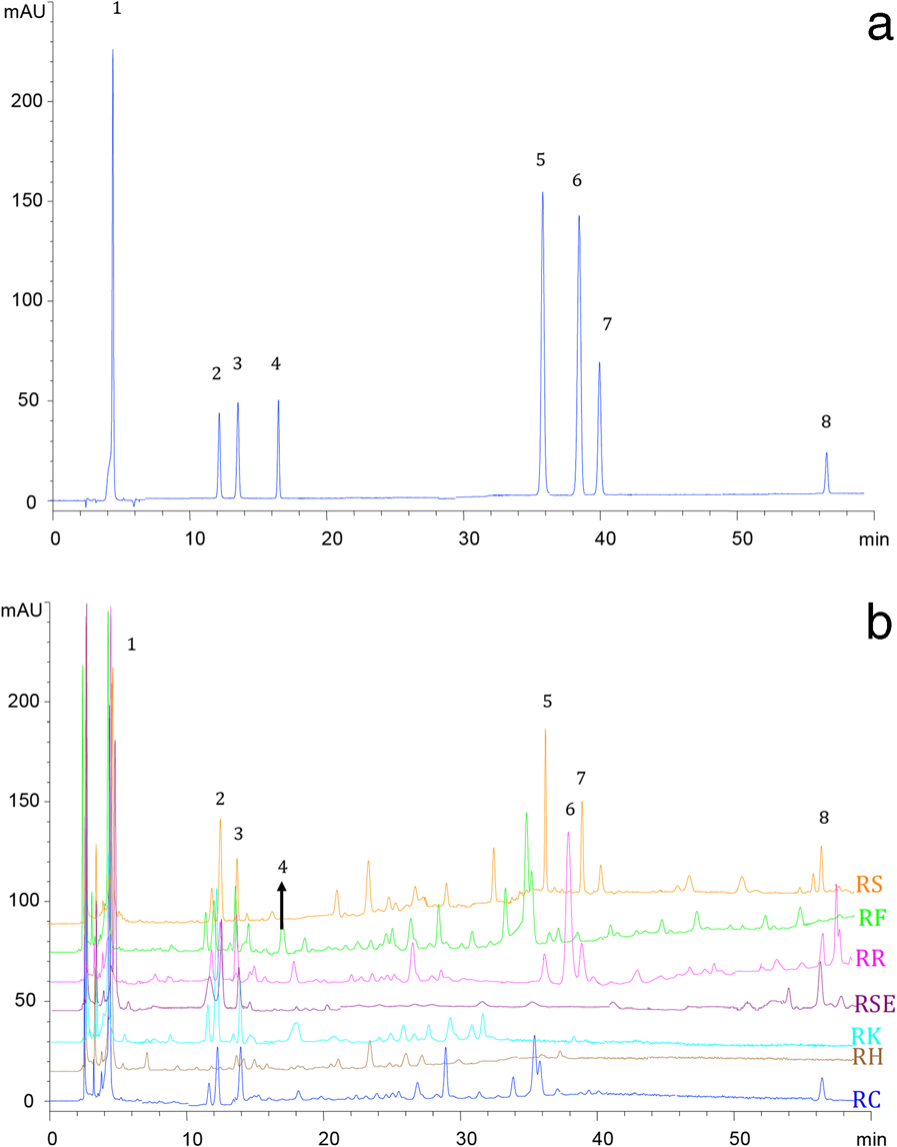
**HPLC-DAD/UV chromatogram of the eight chemotaxonomic markers (a) and overlap chromatogram of each *Rhodiola *species (b).** 1, Gallic acid; 2, Tyrosol; 3, Salidroside; 4, (+) Catechin; 5. Rosarin; 6, Rosavin; 7, Rosin; 8, Rhodionin.

According to the validated method described above, the 8 reference compounds could be visually distinguished from each other, and all the peaks were simultaneously eluted within 55 minutes. The representative chromatograms of each *Rhodiola* species and the eight standard mixtures were provided in Figure 3. It indicated that different kinds of reference markers occurred at various concentrations in the different *Rhodiola* species of Hongjingtian samples. For example, gallic acid and salidroside could be detected in all species. Rosarin and rosin was characteristic in *RS, RH* and *RR* species, which was consistent with the reported literature [5,11]. Rhodionin could barely be detected in *RH* and *RK* species. Rosavin was existed only in *RH, RSE* and *RR* species. The contents of reference compounds were determined for the 47 *Rhodiola* samples, as shown in Table S1 in Additional file 1.

Accordingly, the 23 *Rhodiola* samples classified by genetic taxonomy were analyzed by chemotaxonomic classification using the four types of bioactive compound as reference markers. HCA showed that *Rhodiola* samples were divided into five branches according to their chemotaxonomy (Figure 2b). Of the two classification methods for classifying the genus *Rhodiola* samples, HCA results showed considerably comparable results for both the genotype- and chemotype-based classification methods. This observation may be because different genotypes caused different chemotypes due to the genotype-dependent production of metabolites.

**Figure 4:**
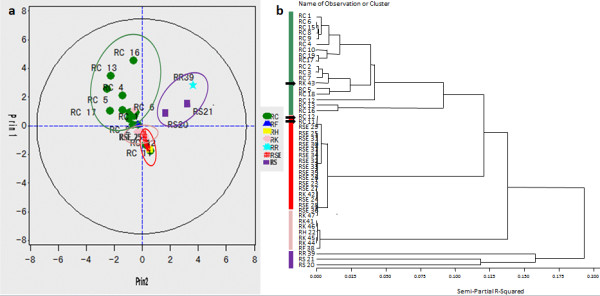
**Principal component analysis (PCA) score plot (a) and hierarchical clustering analysis (HCA) dendrogram (b) derived from the HPLC-DAD/UV data set of the *Rhodiola *samples.**

Furthermore, to differentiate all the *Rhodiola* samples by chemotaxonomic classification, an unsupervised pattern recognition method (PCA) was performed. A two-component PCA score plot of HPLC-DAD/UV data was utilized to depict the general variation of the marker references among the 47 *Rhodiola* samples. As shown in Figure 4a, the samples were primarily divided into four clusters according to their species. The clustering pattern observed from the HCA tree (Figure 4b) is consistent with the PCA. According to the PCA and HCA tree, samples *RK* 43 and *RC* 11 and 12 were distinctively excluded from their original branches, which were merged in the *RC* and *RSE* species, respectively. It indicated that samples of *RK* 43 and *RC* 11 and 12 identified by morphological characteristics are not correct. The present chemotaxonomic classification could be effectively used for *Rhodiola* species identification.

## Conclusions

In this study, the 47 *Rhodiola* samples used commercially in China were identified by their morphological characteristics and genetic and phytochemical taxonomies. In the morphological characteristics, there exists variation between populations of the same species at different life stages and from different environments [29]. If the samples collected are not intact, the accuracy of the identification will be affected. The morphologies of some *Rhodiola* samples are too similar to distinguish visually. Genetic taxonomy can provide exact classification of species submitted to GenBank. However, there may be uncertainty in the extraction of pure DNA from every sample, and the procedure of analyses is time-consuming. Here, eight bioactive compounds from four chemical classes (phenylpropanoids, phenylethanol derivatives, flavonoids and phenolic acids) were used as chemotaxonomic markers to evaluate and distinguish the chemotypes of 47 *Rhodiola* samples by an HPLC-DAD/UV method. First, 23 *Rhodiola* samples classified by genetic taxonomy and morphological characteristics were analyzed by chemotaxonomic classification, which showed considerably comparable results. This analysis indicated that different genotypes caused different chemotypes due to the genotype-dependent production of metabolites. Next, all the 47 *Rhodiola* samples were analyzed by PCA and HCA based on the content of the eight bioactive references. All the samples were divided into four clusters according to the established phytochemical taxonomic method. Consequently, chemotyping became useful for distinguishing morphologically similar species, by identifying variants of the chemotaxonomic markers. However, certain limitations exist in the present studies. The number of collected samples from *RS*, *RH*, *RF* and *RR* species is low, which affected the accuracy of this phytochemical taxonomic classification. Moreover, the clusters of genes for biosynthesis regulating the metabolite profiling need to be investigated in future.

## Competing interests

The authors declare that they have no competing interests.

## Authors’ contributions

LA provided the concept and designed the study. LZ and LY conducted the analyses and wrote the manuscript. LC, SZ, LQ, ZQ, LC, WC, NZ, ZY and TC participated in the research. All authors have read and approved the final manuscript.

## Supplementary Material

Additional file 1 **The DNA sequences and their IDs of the detected samples were provided in the Supplementary Materials.** The contents of reference compounds from 47 *Rhodiola* samples were listed in Table 1S. Click here for file
