# Supplementary material for: The chemotaxonomic classification of Rhodiola plants and its correlation with morphological characteristics and genetic taxonomy
Source: Chem Cent J. 2013 Jul 12;7:118. doi: 10.1186/1752-153X-7-118 (PMC3718703; doi:10.1186/1752-153X-7-118)
Supplement: Additional file 1 — The DNA sequences and their IDs of the detected samples were provided in the Supplementary Materials. The contents of reference compounds from 47 Rhodiola samples were listed in Table 1S. [file 1752-153X-7-118-S1.doc]

***Supporting information：***

**The chemotaxonomic classification of *Rhodiola* plants and its correlation with morphological characteristics and genetic taxonomy**

Zhenli Liu**^§^**^1^, Yuanyan Liu**^§^**^2^, Chunsheng Liu^2^, Zhiqian Song^1^, Qing Li^1^, Qinglin Zha^3^,

Cheng Lu^3^, Cheng Tian^2^, Zhangchi Ning^2^, Yuxin Zhang ^2^ Aiping Lu3, 4[[1]](#footnote-2)*

**Affiliation**

^1^ Institution of Basic Theory, China Academy of Chinese Medical Sciences, Beijing, China

^2^ School of Chinese Materia Medica, Beijing University of Chinese Medicine, Beijing Municipal Key Laboratory for Basic Research of Chinese Medicine, Beijing, China

^3^ Institute of Basic Research in Clinical Medicine, China Academy of Chinese Medical Sciences, Beijing, China

^4^ School of Chinese Medicine, Hong Kong Baptist University, Kowloon Tong, Hong Kong SAR, China

**H1** Alignment of the deduced amino acid sequences of gene of H1 (a) and GenBank ([EU239666.1](http://www.ncbi.nlm.nih.gov/nucleotide/159795884?report=genbank&log$=nucltop&blast_rank=1&RID=ZC4GHC4C01S)) (b).

**a.**

1 TTTCCGTAGG TGAACCTGCG GAAGGATCAT TGTCGAAGCC TAGAAAGCAT GACCGTGGAC

61 AAGTTGTTAA TCGTGGATGC ACTTGGATCC TTCGGGGTAC TCCATTTGCC TTCCACTCGG

121 TCGGTGATGA GCCTTGCGCC CATCGCTGAT TGAAACGAAT GAACCCCGGC GCGGATCGCG

181 CCAAGGAATC GAAACCGGAA GAGCTTGGCT CGGTGGCCCG TTCTCGGGCC GCCAAGGCCT

241 TAGGTCTATT CAAAATACCA TAACGACTCT CGGCAACGGA TATCTCGGCT CTCGCATCGA

301 TGAAGAACGT AGCAAAATGC GATACTTGGT GTGAATTGCA GAATCCCGTG AACCATCGAG

361 TTTTTGAACG CAAGTTGCGC CCGAAGCCAT TAGGCCGAGG GCACGTCTGC CTGGGCGTCA

421 CGTATCGTGT TGCCCCCATA CATGTATTGG GGGTGAAGCT TGGCCTCCCG TGAGCCCAAA

481 CTCGCGGATG GCTTAAAAAC GAGCCTCGAG ACGGTTAGAC GTCGCGACAA GTGGTGGTTA

541 CGAGGCCTTG CGCCTTTGAG CTTTGCGCGT CGCTGCCGTC GTCCCTCTCT CTCGAAATTA

601 TAACCCGAAC GGAGCATCCT TGATGTTTCC AGCATTGCGA CCCCAGGTCA GGCGGGAGTA

661 CCCGCTGAGT TTAA

**b.**


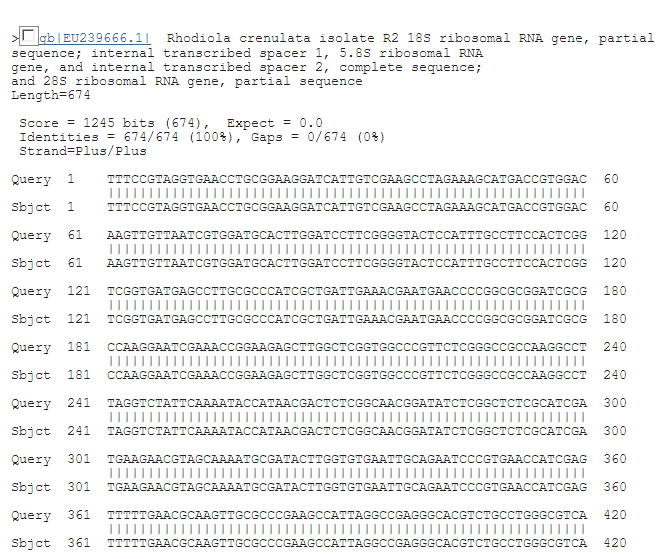

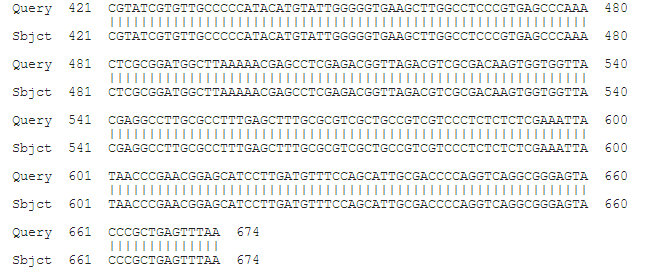


**H2** Alignment of the deduced amino acid sequences of gene of H1 (a) and GenBank ([EU239666.1](http://www.ncbi.nlm.nih.gov/nucleotide/159795884?report=genbank&log$=nucltop&blast_rank=1&RID=ZC4GHC4C01S)) (b).

**a.**

1 TTTCCGTAGG TGAACCTGCG GAAGGATCAT TGTCGAAGCC TAGAAAGCAT GACCGTGGAC

61 AAGTTGTTAA TCGTGGATGC ACTTGGATCC TTCGGGGTAC TCCATTTGCC TTCCACTCGG

121 TCGGTGATGA GCCTTGCGCC CATCGCTGAT TGAAACGAAT GAACCCCGGC GCGGATCGCG

181 CCAAGGAATC GAAACCGGAA GAGCTTGGCT CGGTGGCCCG TTCTCGGGCC GCCAAGGCCT

241 TAGGTCTATT CAAAATACCA TAACGACTCT CGGCAACGGA TATCTCGGCT CTCGCATCGA

301 TGAAGAACGT AGCAAAATGC GATACTTGGT GTGAATTGCA GAATCCCGTG AACCATCGAG

361 TTTTTGAACG CAAGTTGCGC CCGAAGCCAT TAGGCCGAGG GCACGTCTGC CTGGGCGTCA

421 CGTATCGTGT TGCCCCCATA CATGTATTGG GGGTGAAGCT TGGCCTCCCG TGAGCCCAAA

481 CTCGCGGATG GCTTAAAAAC GAGCCTCGAG ACGGTTAGAC GTCGCGACAA GTGGTGGTTA

541 CGAGGCCTTG CGCCTTTGAG CTTTGCGCGT CGCTGCCGTC GTCCCTCTCT CTCGAAATTA

601 TAACCCGAAC GGAGCATCCT TGATGTTTCC AGCATTGCGA CCCCAGGTCA GGCGGGAGTA

661 CCCGCTGAGT TTAA

**b.**


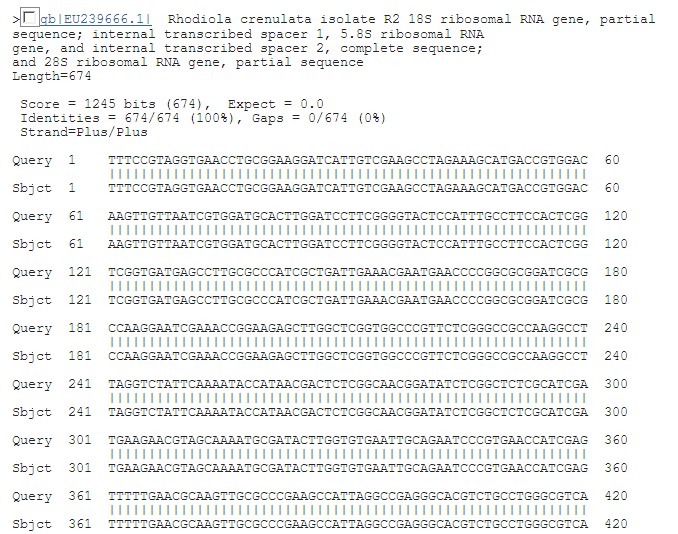

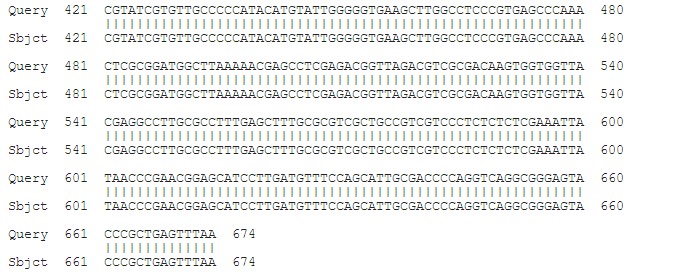


**H3** Alignment of the deduced amino acid sequences of gene of H1 (a) and GenBank ([EU239666.1](http://www.ncbi.nlm.nih.gov/nucleotide/159795884?report=genbank&log$=nucltop&blast_rank=1&RID=ZC4GHC4C01S)) (b).

**a.**

1 TTTCCGTAGG TGAACCTGCG GAAGGATCAT TGTCGAAGCC TAGAAAGCAT GACCGTGGAC

61 AAGTTGTTAA TCGTGGATGC ACTTGGATCC TTCGGGGTAC TCCATTTGCC TTCCACTCGG

121 TCGGTGATGA GCCTTGCGCC CATCGCTGAT TGAAACGAAT GAACCCCGGC GCGGATCGCG

181 CCAAGGAATC GAAATCGGAA GAGCTTGGCT CGGTGGCCCG TTCTCGGGCC GCCAAGGCCT

241 TAGGTCTATT CAAAATACCA TAACGACTCT CGGCAACGGA TATCTCGGCT CTCGCATCGA

301 TGAAGAACGT AGCAAAATGC GATACTTGGT GTGAATTGCA GAATCCCGTG AACCATCGAG

361 TTTTTGAACG CAAGTTGCGC CCGAAGCCAT TAGGCCGAGG GCACGTCTGC CTGGGCGTCA

421 CGTATCGTGT TGCCCCCATA CATGTATTGG GGGTGAAGCT TGGCCTCCCG TGAGCCCAAA

481 CTCGCGGATG GCTTAAAAAC GAGCCTCGAG ACGGTTAGAC GTCGCGACAA GTGGTGGTTA

541 CGAGGCCTTG CGCCTTTGAG CTTTGCGCGT CGCTGCCGTC GTCCCTCTCT CTCGAAATTA

601 TAACCCGAAC GGAGCATCCT TGATGTTTCC AGCATTGCGA CCCCAGGTCA GGCGGGAGTA

661 CCCGCTGAGT TTAA

**b.**


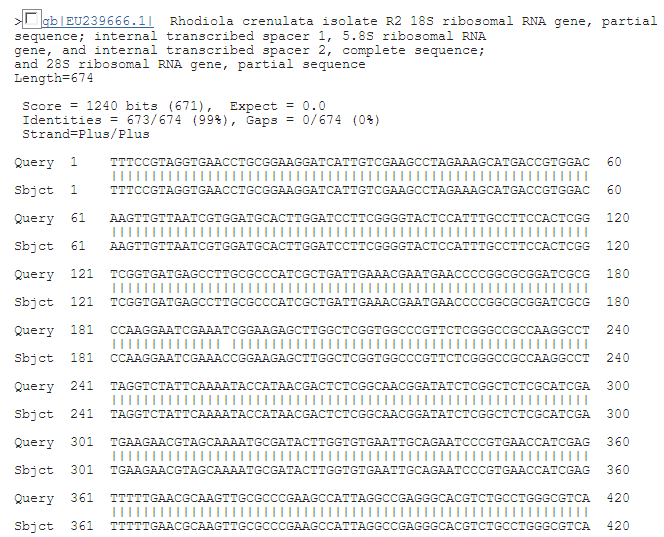

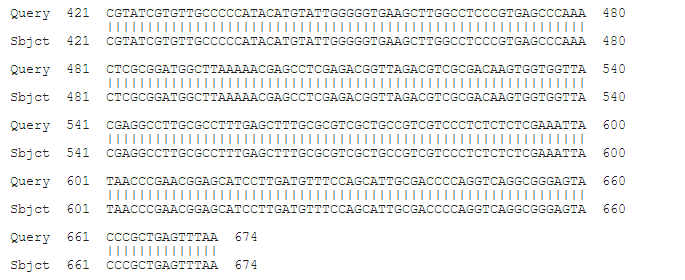


**H4** Alignment of the deduced amino acid sequences of gene of H1 (a) and GenBank ([EU239666.1](http://www.ncbi.nlm.nih.gov/nucleotide/159795884?report=genbank&log$=nucltop&blast_rank=1&RID=ZC4GHC4C01S)) (b).

**a.**

1 TTTCCGTAGG TGAACCTGCG GAAGGATCAT TGTCGAAGCC TAGAAAGCAT GACCGTGGAC

61 AAGTTGTTAA TCGTGGATGC ACTTGGATCC TTCGGGGTAC TCCATTTGCC TTCCACTCGG

121 TCGGTGATGA GCCTTGCGCC CATCGCTGAT TGAAACGAAT GAACCCCGGC GCGGATCGCG

181 CCAAGGAATC GAAACCGGAA GAGCTTGGCT CGGTGGCCCG TTCTCGGGCC GCCAAGGCCT

241 TAGGTCTATT CAAAATACCA TAACGACTCT CGGCAACGGA TATCTCGGCT CTCGCATCGA

301 TGAAGAACGT AGCAAAATGC GATACTTGGT GTGAATTGCA GAATCCCGTG AACCATCGAG

361 TTTTTGAACG CAAGTTGCGC CCGAAGCCAT TAGGCCGAGG GCACGTCTGC CTGGGCGTCA

421 CGTATCGTGT TGCCCCCATA CATGTATTGG GGGTGAAGCT TGGCCTCCCG TGAGCCCAAA

481 CTCGCGGATG GCTTAAAAAC GAGCCTCGAG ACGGTTAGAC GTCGCGACAA GTGGTGGTTA

541 CGAGGCCTTG CGCCTTTGAG CTTTGCGCGT CGCTGCCGTC GTCCCTCTCT CTCGAAATTA

601 TAACCCGAAC GGAGCATCCT TGATGTTTCC AGCATTGCGA CCCCAGGTCA GGCGGGAGTA

661 CCCGCTGAGT TTAA

**b.**


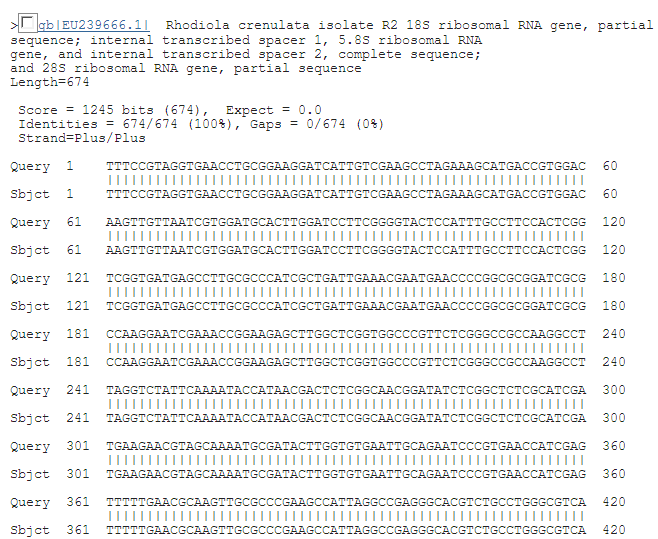

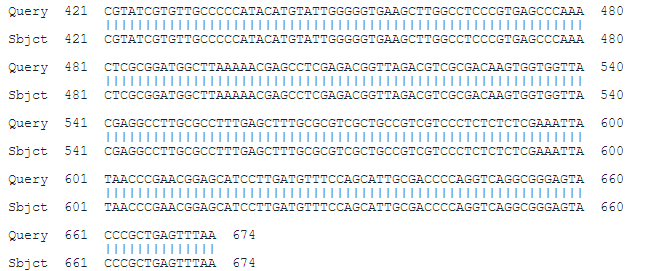


**H6** Alignment of the deduced amino acid sequences of gene of H1 (a) and GenBank ([EU239666.1](http://www.ncbi.nlm.nih.gov/nucleotide/159795884?report=genbank&log$=nucltop&blast_rank=1&RID=ZC4GHC4C01S)) (b).

**a. H6-1**

TTTCCGTAGG TGAACCTGCG GAAGGATCAT TGTCGAAGCC TAGAAAGCAT GACCGTGGAC

61 AAGTTGTTAA TCGTGGATGC ACTTGGATCC TTCGGGGTAC TCCATTTGCC TTCCACTCGG

121 TCGGTGATGA GCCTTGCGCC CATCGCTGAT TGAAACGAAT GAACCCCGGC GCGGATCGCG

181 CCAAGGAATC GAAACCGGAA GAGCTTGGCT CGGTGGCCCG TTCTCGGGCC GCCAAGGCCT

241 TAGGTCTATT CAAAATACCA TAACGACTCT CGGCAACGGA TATCTCGGCT CTCGCATCGA

301 TGAAGAACGT AGCAAAATGC GATACTTGGT GTGAATTGCA GAATCCCGTG AACCATCGAG

361 TTTTTGAACG CAAGTTGCGC CCGAAGCCAT TAGGCCGAGG GCACGTCTGC CTGGGCGTCA

421 CGTATCGTGT TGCCCCCATA CATGTATTGG GGGTGAAGCT TGGCCTCCCG TGAGCCCAAA

481 CTCGCGGATG GCTTAAAAAC GAGCCTCGAG ACGGTTAGAC GTCGCGACAA GTGGTGGTTA

541 CGAGGCCTTG CGCCTTTGAG CTTTGCGCGT CGCTGCCGTC GTCCCTCTCT CTCGAAATTA

601 TAACCCGAAC GGAGCATCCT CGATGTTTCC AGCATTGCGA CCCCAGGTCA GGCGGGAGTA

661 CCCGCTGAGT TTAA

**H6-2**

1 TTTCCGTAGG TGAACCTGCG GAAGGATCAT TGTCGAAGCC TAGAAAGCAT GACCGTGGAC

61 AAGTTGTTAA TCGTGGATGC ACTTGGATCC TTCGGGGTAC TCCATTTGCC TTCCACTCGG

121 TCGGTGATGA GCCTTGCGCC CATCGCTGAT TGAAACGAAT GAACCCCGGC GCGGATCGCG

181 CCAAGGAATC GAAATCGGAA GAGCTTGGCT CGGTGGCCCG TTCTCGGGCC GCCAAGGCCT

241 TAGGTCTATT CAAAATACCA TAACGACTCT CGGCAACGGA TATCTCGGCT CTCGCATCGA

301 TGAAGAACGT AGCAAAATGC GATACTTGGT GTGAATTGCA GAATCCCGTG AACCATCGAG

361 TTTTTGAACG CAAGTTGCGC CCGAAGCCAT TAGGCCGAGG GCACGTCTGC CTGGGCGTCA

421 CGTATCGTGT TGCCCCCATA CATGTATTGG GGGTGAAGCT TGGCCTCCCG TGAGCCCAAA

481 CTCGCGGATG GCTTAAAAAC GAGCCTCGAG ACGGTTAGAC GTCGCGACAA GTGGTGGTTA

541 CGAGGCCTTG CGCCTTTGAG CTTTGCGCGT CGCTGCCGTC GTCCCTCTCT CTCGAAATTA

601 TAACCCGAAC GGAGCATCCT TGATGTTTCC AGCATTGCGA CCCCAGGTCA GGCGGGAGTA

661 CCCGCTGAGT TTAA

**b. H6-1**


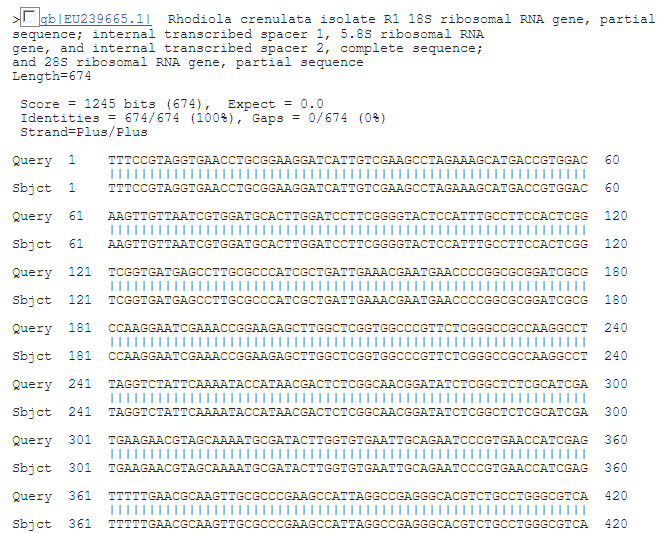

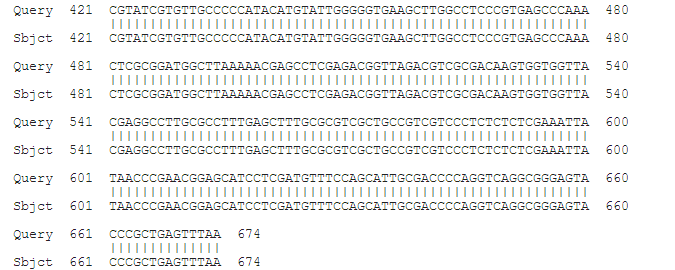


**H6-2**


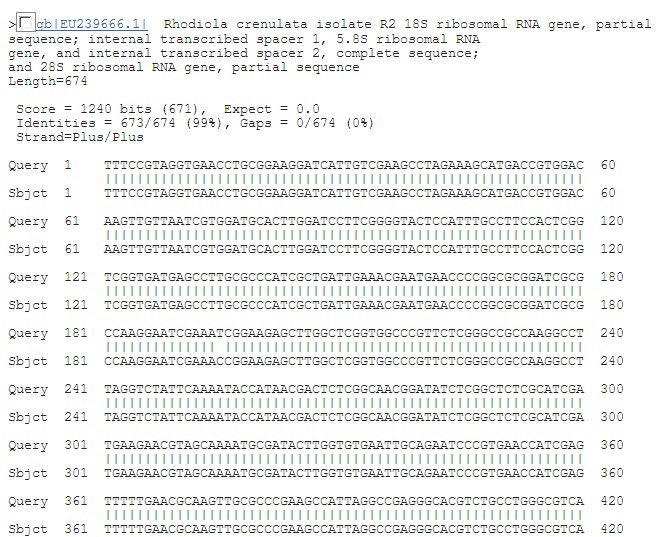

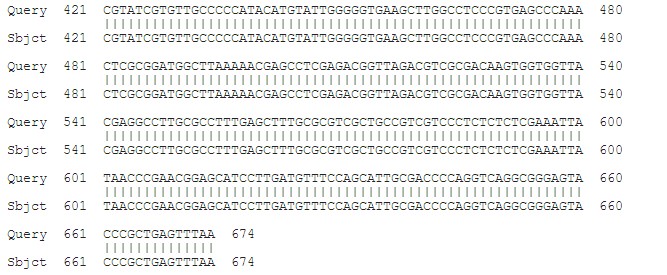


**H7** Alignment of the deduced amino acid sequences of gene of H1 (a) and GenBank ([EU239666.1](http://www.ncbi.nlm.nih.gov/nucleotide/159795884?report=genbank&log$=nucltop&blast_rank=1&RID=ZC4GHC4C01S)) (b).

**a.**

1 TTTCCGTAGG TGAACCTGCG GAAGGATCAT TGTCGAAGCC TAGAAAGCAT GACCGTGGAC

61 AAGTTGTTAA TCGTGGATGC ACTTGGATCC TTCGGGGTAC TCCATTTGCC TTCCACTCGG

121 TCGGTGATGA GCCTTGCGCC CATCGCTGAT TGAAACGAAT GAACCCCGGC GCGGATCGCG

181 CCAAGGAATC GAAACCGGAA GAGCTTGGCT CGGTGGCCCG TTCTCGGGCC GCCAAGGCCT

241 TAGGTCTATT CAAAATACCA TAACGACTCT CGGCAACGGA TATCTCGGCT CTCGCATCGA

301 TGAAGAACGT AGCAAAATGC GATACTTGGT GTGAATTGCA GAATCCCGTG AACCATCGAG

361 TTTTTGAACG CAAGTTGCGC CCGAAGCCAT TAGGCCGAGG GCACGTCTGC CTGGGCGTCA

421 CGTATCGTGT TGCCCCCATA CATGTATTGG GGGTGAAGCT TGGCCTCCCG TGAGCCCAAA

481 CTCGCGGATG GCTTAAAAAC GAGCCTCGAG ACGGTTAGAC GTCGCGACAA GTGGTGGTTA

541 CGAGGCCTTG CGCCTTTGAG CTTTGCGCGT CGCTGCCGTC GTCCCTCTCT CTCGAAATTA

601 TAACCCGAAC GGAGCATCCT TGATGTTTCC AGCATTGCGA CCCCAGGTCA GGCGGGAGTA

661 CCCGCTGAGT TTAA

**b.**


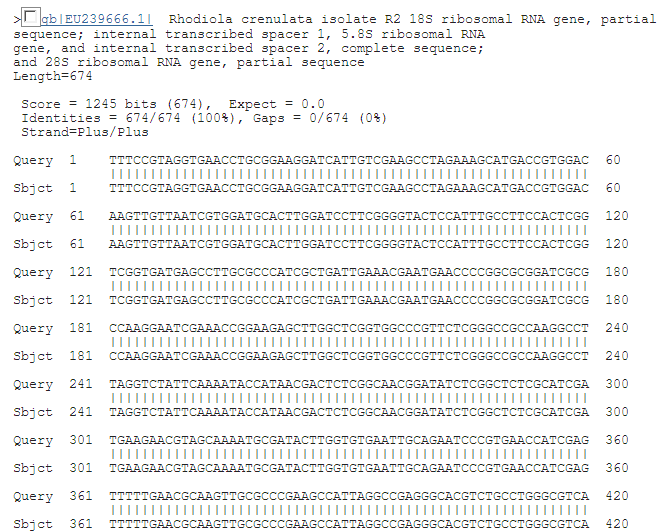

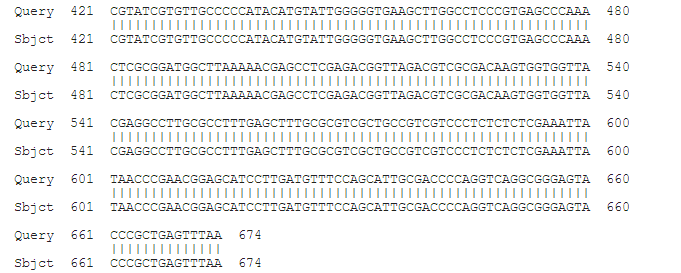


**H8** Alignment of the deduced amino acid sequences of gene of H1 (a) and GenBank ([AB088597.1](http://www.ncbi.nlm.nih.gov/nucleotide/26522883?report=genbank&log$=nucltop&blast_rank=1&RID=ZDJ1MFPY012)) (b).

**a.**

1 TTTCCGTAGG TGAACCTGCG GAAGGATCAT TGTCGAAGCC TAGAAAGCAT GACCGTGGAC

61 AAGTTGTTAA TCGTGGATGC ACTTGGATCC TTCGGGGTAC TCCATTTGCC TTCCACTCGG

121 TCGGTGATGA GCCTTGCGCC CATCGCTGAT TGAAACGAAT GAACCCCGGC GCGGATCGCG

181 CCAAGGAATC GAAACCGGAA GAGCTTGGCT CGGTGGCCCG TTCTCGGGCT GCCAAGGCCT

241 TAGGTCTATT CAAAATACCA TAACGACTCT CGGCAACGGA TATCTCGGCT CTCGCATCGA

301 TGAAGAACGT AGCAAAATGC GATACTTGGT GTGAATTGCA GAATCCCGTG AACCATCGAG

361 TTTTTGAACG CAAGTTGCGC CCGAAGCCAT TAGGCCGAGG GCACGTCTGC CTGGGCGTCA

421 CGTATCGTGT TGCCCCCATA CATGTATTGG GGGTGAAGCT TGGCCTCCCG TGAGCCCAAA

481 CTCGCGGATG GCTTAAAAAC GAGCCTCGAG ACGGCTAGAC GTCGCGATAA GTGGTGGTTG

541 CAAGGCCTTG TGCCTTTGAG CGTTGCGCGT CGCTGCCGTC GTCCCTCTCT CTCGAAATTA

601 TAACCCGAAC GGAGCATCCT TGATGTTTCC AGCATTGCGA CCCCAGGTCA GGCGGGAGTA

661 CCCGCTGAGT TTAA

**b.**


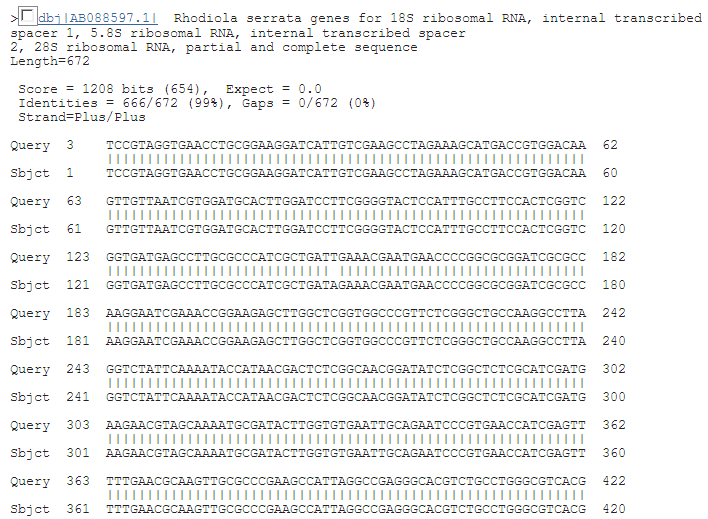

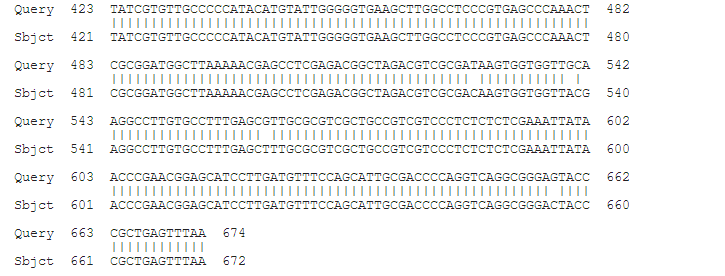


**H9** Alignment of the deduced amino acid sequences of gene of H1 (a) and GenBank ([EU239665.1](http://www.ncbi.nlm.nih.gov/nucleotide/159795883?report=genbank&log$=nucltop&blast_rank=1&RID=ZEP95SP601N)) (b).

**a.**

1 TTTCCGTAGG TGAACCTGCG GAAGGATCAT TGTCGAAGCC TAGAAAGCAT GACCGTGGAC

61 AAGTTGTTAA TCGTGGATGC ACTTGGATCC TTCGGGGTAC TCCATTTGCC TTCCACTCGG

121 TCGGTGATGA GCCTTGCGCC CATCGCTGAT TGAAACGAAT GAACCCCGGC GCGGATCGCG

181 CCAAGGAATC GAAACCGGAA GAGCTTGGCT CGGGGGCCCG TTCTCGGGCC GCCAAGGCCT

241 TAGGTCTATT CAAAATACCA TAACGACTCT CGGCAACGGA TATCTCGGCT CTCGCATCGA

301 TGAAGAACGT AGCAAAATGC GATACTTGGT GTGAATTGCA GAATCCCGTG AACCATCGAG

361 TTTTTGAACG CAAGTTGCGC CCGAAGCCAT TAGGCCGAGG GCACGTCTGC CTGGGCGTCA

421 CGTATCGTGT TGCCCCCATA CATGTATTGG GGGTGAAGCT TGGCCTCCCG TGAGCCCAAA

481 CTCGCGGATG GCTTAAAAAC GAGCCTCGAG ACGGTTAGAC GTCGCGACAA GTGGTGGTTA

541 CGAGGCCTTG CGCCTTTGAG CTTTGCGCGT CGCTGCCGTC ATCCCTCTCT CTCGAAATTA

601 TAACCCGAAC GGATCATCCT CGATGTTTCC AGCATTGCGA CCTCAGGTCA GGCGGGAGTA

661 CCCGCTGAGT TTAA

**b.**


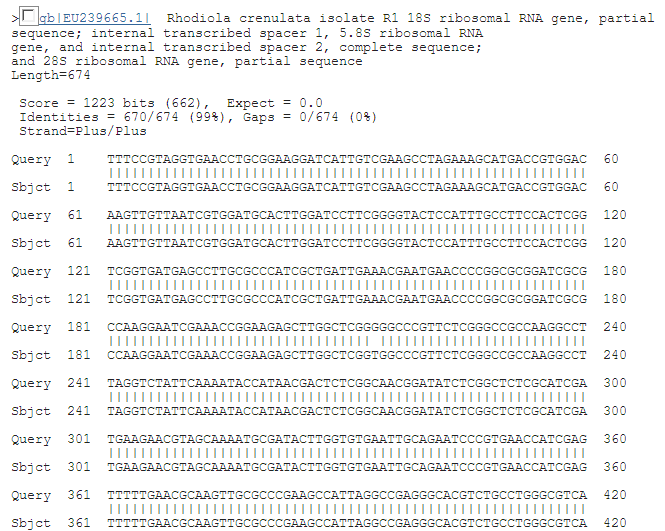

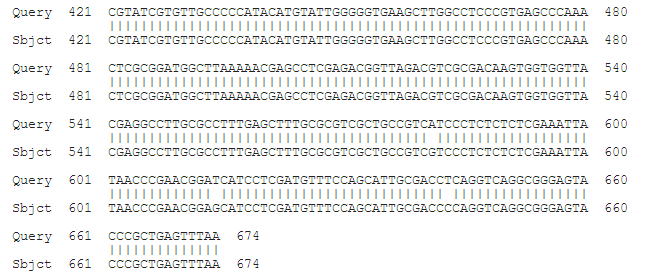


**H10** Alignment of the deduced amino acid sequences of gene of H1 (a) and GenBank ([AB088597.1](http://www.ncbi.nlm.nih.gov/nucleotide/26522883?report=genbank&log$=nucltop&blast_rank=1&RID=ZDJ1MFPY012)) (b).

**a.**

H10-1 TTTCCGTAGGTGAACCTGCGGAAGGATCATTGTCGAAGCCTAGAAAGCAT 50

H10-2 -------------------------------------------------- 50

H10-1 GACCGTGGACAAGTTGTTAATCGTGGATGCACTTGGATCCTTCGGGGTAC 100

H10-2 -------------------------------------------------- 100

H10-1 TCCATTTGCCTTCCACTCGGTCGGTGATGAGCCTTGCGCCCATCGCTGAT 150

H10-2 ---------------------t---------------------------- 150

H10-1 TGAAACGAATGAACCCCGGCGCGGATCGCGCCAAGGAATCGAAACCGGAA 200

H10-2 -------------------------------------------------- 200

H10-1 GAGCTTGGCTCGGTGGCCCGTTCTCGGGCTGCCAAGGCCTTAGGTCTATT 250

H10-2 -------------------------------------------------- 250

H10-1 CAAAATACCATAACGACTCTCGGCAACGGATATCTCGGCTCTCGCATCGA 300

H10-2 -------------------------------------------------- 300

H10-1 TGAAGAACGTAGCAAAATGCGATACTTGGTGTGAATTGCAGAATCCCGTG 350

H10-2 -------------------------------------------------- 350

H10-1 AACCATCGAGTTTTTGAACGCAAGTTGCGCCCGAAGCCATTAGGCCGAGG 400

H10-2 -------------------------------------------------- 400

H10-1 GCACGTCTGCCTGGGCGTCACGTATCGTGTTGCCCCCATACATGTATTGG 450

H10-2 -------------------------------------------------- 450

H10-1 GGGTGAAGCTTGGCCTCCCGTGAGCCCAAACTCGCGGATGGCTTAAAAAC 500

H10-2 -------------------------------------------------- 500

H10-1 GAGCCTCGAGACGGCTAGACGTCGCGATAAGTGGTGGTTGCAAGGCCTTG 550

H10-2 -------------------------------------------------- 550

H10-1 TGCCTTTGAGCGTTGCGCGTCGCTGCCGTCGTCCCTCTCTCTCGAAATTA 600

H10-2 -------------------------------------------------- 600

H10-1 TAACCCGAACGGAGCATCCTTGATGTTTCCAGCATTGCGACCCCAGGTCA 650

H10-2 -------------------------------------------------- 650

H10-1 GGCGGGAGTACCCGCTGAGTTTAA 674

H10-2 ------------------------ 674

**b. H10-1**


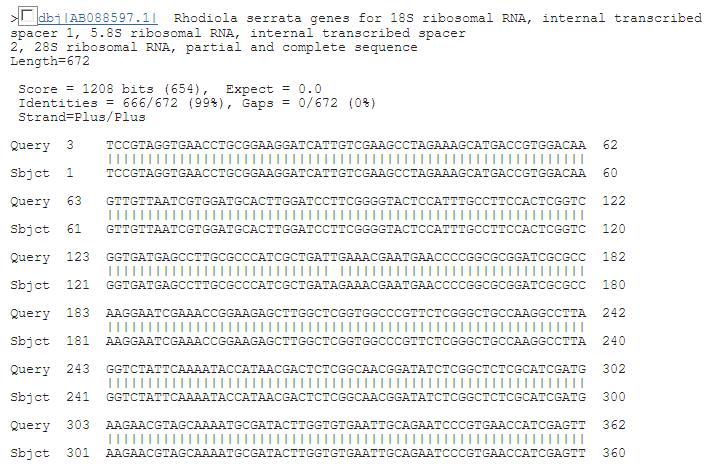

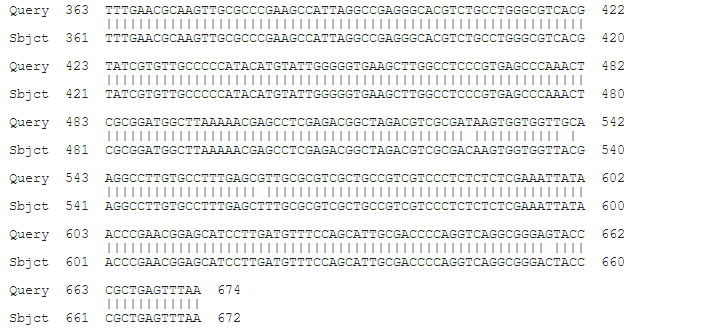


**H10-2**


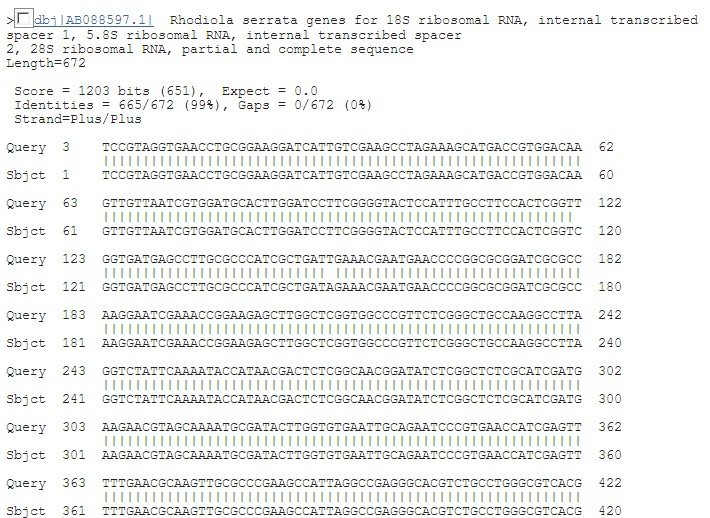


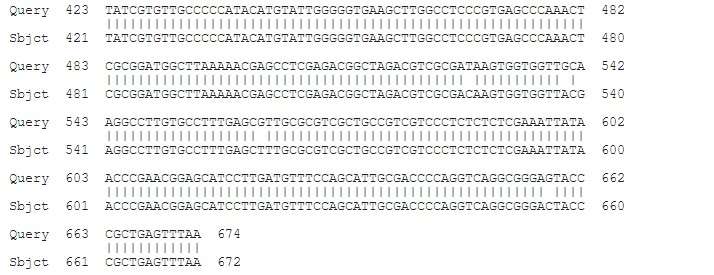


**H11** Alignment of the deduced amino acid sequences of gene of H1 (a) and GenBank ([AB088597.1](http://www.ncbi.nlm.nih.gov/nucleotide/26522883?report=genbank&log$=nucltop&blast_rank=1&RID=ZDJ1MFPY012)) (b).

**a.**

1 TTTCCGTAGG TGAACCTGCG GAAGGATCAT TGTCGAAGCC TAGAAAGCAT GACCGTGGAC

61 AAGTTGTTAA TCGTGGATGC ACTTGGATCC TTCGGGGTAC TCCATTTGCC TTCCACTCGG

121 TCGGTGATGA GCCTTGCGCC CATCGCTGAT TGAAACGAAT GAACCCCGGC GCGGATCGCG

181 CCAAGGAATC GAAACCGGAA GAGCTTGGCT CGGTGGCCCG TTCTCGGGCT GCCAAGGCCT

241 TAGGTCTATT CAAAATACCA TAACGACTCT CGGCAACGGA TATCTCGGCT CTCGCATCGA

301 TGAAGAACGT AGCAAAATGC GATACTTGGT GTGAATTGCA GAATCCCGTG AACCATCGAG

361 TTTTTGAACG CAAGTTGCGC CCGAAGCCAT TAGGCCGAGG GCACGTCTGC CTGGGCGTCA

421 CGTATCGTGT TGCCCCCATA CATGTATTGG GGGTGAAGCT TGGCCTCCCG TGAGCCCAAA

481 CTCGCGGATG GCTTAAAAAC GAGCCTCGAG ACGGCTAGAC GTCGCGATAA GTGGTGGTTG

541 CAAGGCCTTG TGCCTTTGAG CGTTGCGCGT CGCTGCCGTC GTCCCTCTCT CTCGAAATTA

601 TAACCCGAAC GGAGCATCCT TGATGTTTCC AGCATTGCGA CCCCAGGTCA GGCGGGAGTA

661 CCCGCTGAGT TTAA

**b.**


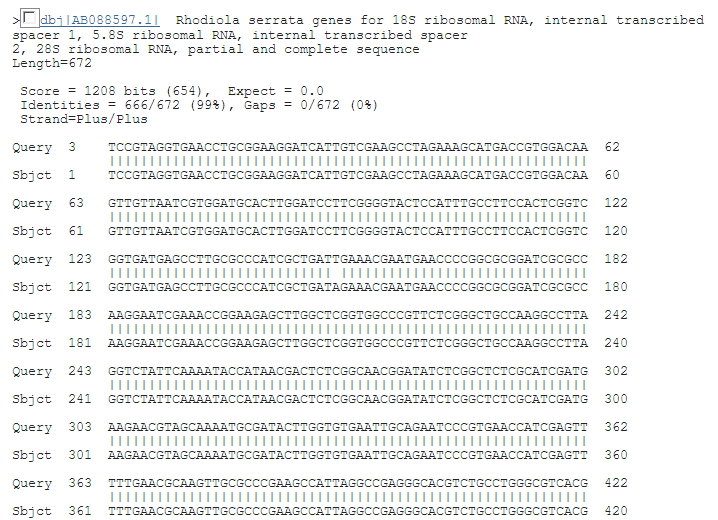

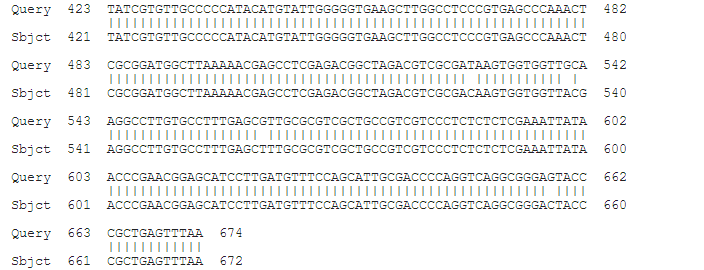


**H13** Alignment of the deduced amino acid sequences of gene of H1 (a) and GenBank ([AB088593.1](http://www.ncbi.nlm.nih.gov/nucleotide/26522870?report=genbank&log$=nucltop&blast_rank=1&RID=ZEGHEU90014)) (b).

**a.**

1 TTTCCGTAGG TGAACCTGCG GAAGGATCAT TGTCGAAGCC TAGAAAGCAT GACCGCGGAC

61 AAGTTGTTAA TCGTGGATGC ACTTGGATCC TTCGGGTACT CCATTTGCCT TCCCCTCGGT

121 CGGTGATGAG CCTTGCGCGC ATTGCTGATC GAAACGAATG AACCCCGGCG CGGATCGCGC

181 CAAGGAATCA AAACCGAAAG AGCTTAGCTC GGTGGCCCGT TCTCGGGTTG CCAGGGCCAT

241 AGGTCTATTC AAAATACCAT AACGACTCTC GGCAACGGAT ATCTCGGCTC TCGCATCGAT

301 GAAGAACGTA GCAAAATGCG ATACTTGGTG TGAATTGCAG AATCCCGTGA ACCATCGAGT

361 TTTTGAACGC AAGTTGCGCC CGAAGCCATT AGGCCGAGGG CACGTCTGCC TGGGCGTCAC

421 ATATTGTGTT GCCCCCATAC ATGTATTGGG GGCGAAGCTT GGCCTACCGT GAGCCCAAAC

481 TCGCGGATGG CTTAAAAACG AGCCTCGAGA CTGTTAAACG TCGCGATAAG TGGTGGTTAC

541 AAGGCCTTGT GCCTTTGAGC GTTGCGTGTC GCTGCCGTCG TCCCTCTCTC TCGAAATTAT

601 AACCCGAAGG GAGCATCCTC GATGTTTCCA GCATTGCGAC CCCAGGTCAG GCGGGACTAC

661 CCGCTGAGTT TAA

**b.**


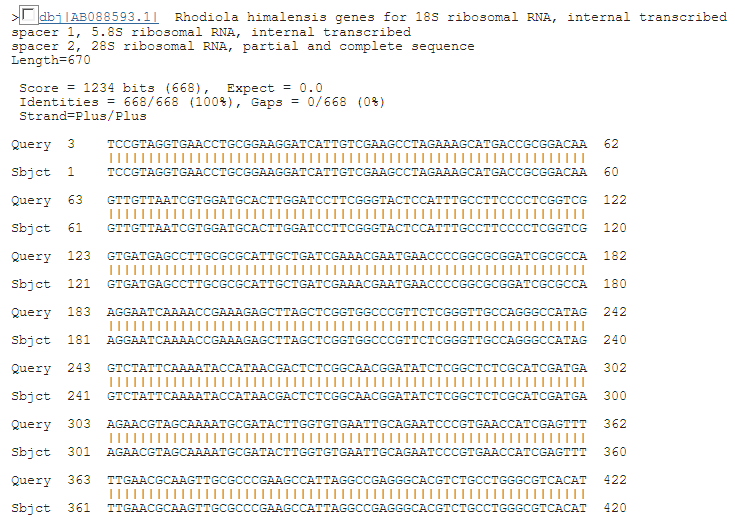

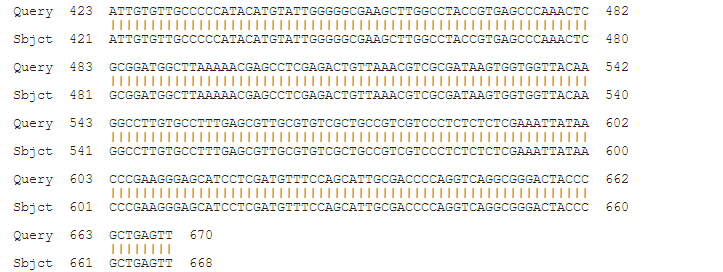


**H14** Alignment of the deduced amino acid sequences of gene of H1 (a) and GenBank ([AB088597.1](http://www.ncbi.nlm.nih.gov/nucleotide/26522883?report=genbank&log$=nucltop&blast_rank=1&RID=ZDJ1MFPY012)) (b).

**a.**

1 TTTCCGTAGG TGAACCTGCG GAAGGATCAT TGTCGAAGCC TAGAAAGCAT GACCGTGGAC

61 AAGTTGTTAA TCGTGGATGC ACTTGGATCC TTCGGGGTAC TCCATTTGCC TTCCACTCGG

121 TCGGTGATGA GCCTTGCGCC CATCGCTGAT TGAAACGAAT GAACCCCGGC GCGGATCGCG

181 CCAAGGAATC GAAACCGGAA GAGCTTGGCT CGGTGGCCCG TTCTCGGGCT GCCAAGGCCT

241 TAGGTCTATT CAAAATACCA TAACGACTCT CGGCAACGGA TATCTCGGCT CTCGCATCGA

301 TGAAGAACGT AGCAAAATGC GATACTTGGT GTGAATTGCA GAATCCCGTG AACCATCGAG

361 TTTTTGAACG CAAGTTGCGC CCGAAGCCAT TAGGCCGAGG GCACGTCTGC CTGGGCGTCA

421 CGTATCGTGT TGCCCCCATA CATGTATTGG GGGTGAAGCT TGGCCTCCCG TGAGCCCAAA

481 CTCGCGGATG GCTTAAAAAC GAGCCTCGAG ACGGCTAGAC GTCGCGATAA GTGGTGGTTG

541 CAAGGCCTTG TGCCTTTGAG CGTTGCGCGT CGCTGCCGTC GTCCCTCTCT CTCGAAATTA

601 TAACCCGAAC GGAGCATCCT TGATGTTTCC AGCATTGCGA CCCCAGGTCA GGCGGGAGTA

661 CCCGCTGAGT TTAA

**b.**


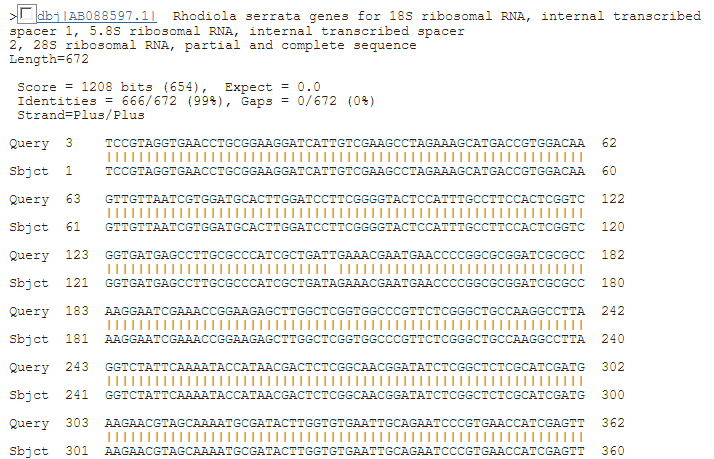

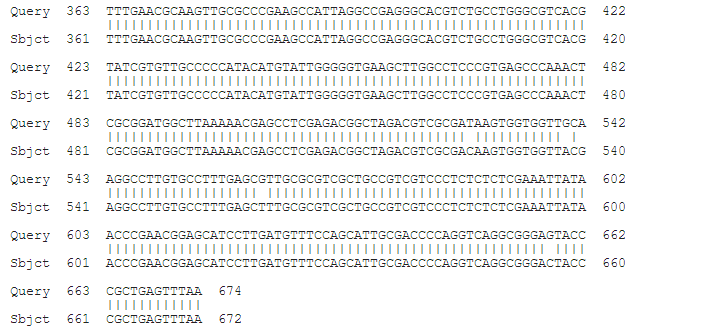


**H16** Alignment of the deduced amino acid sequences of gene of H1 (a) and GenBank (AB088597.1) (b).

**a.**

TTTCCGTA GGTGAACCTG CGGAAGGATC ATTGTCGAAG CCTAGAAAGC ATGACCGTGG ACAAGTTGTT AATCGTGGAT GCACTTGGAT CCTTCGGGGT ACTCCATTTG CCTTCCACTC GGTCGGTGAT GAGCCTTGCG CCCATCGCTG ATTGAAACGA ATGAACCCCG GCGCGGATCG CGCCAAGGAA TCGAAACCGG AAGAGCTTGG CTCGGTGGCC CGTTCTCGGG CTGCCAAGGC CTTAGGTCTA TTCAAAATAC CATAACGACT CTCGGCAACG GATATCTCGG CTCTCGCATC GATGAAGAAC GTAGCAAAAT GCGATACTTG GTGTGAATTG CAGAATCCCG TGAACCATCG AGTTTTTGAA CGCAAGTTGC GCCCGAAGCC ATTAGGCCGA GGGCACGTCT GCCTGGGCGT CACGTATCGT GTTGCCCCCA TACATGTATT GGGGGTGAAG CTTGGCCTCC CGTGAGCCCA AACTCGCGGA TGGCTTAAAA ACGAGCCTCG AGACGGCTAG ACGTCGCGAT AAGTGGTGGT TGCAAGGCCT TGTGCCTTTG AGCGTTGCGC GTCGCTGCCG TCGTCCCTCT CTCTCGAAAT TATAACCCGA ACGGAGCATC CTTGATGTTT CCAGCATTGC GACCCCAGGT CAGGCGGGAG TACCCGCTGA GTTTAA

**b.**


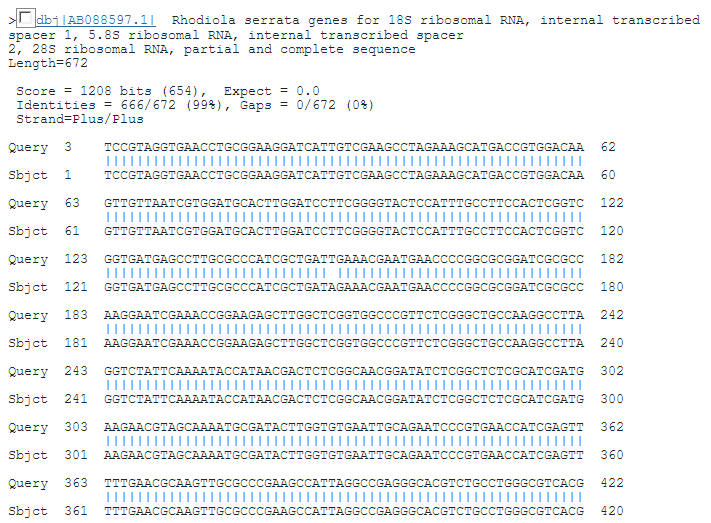


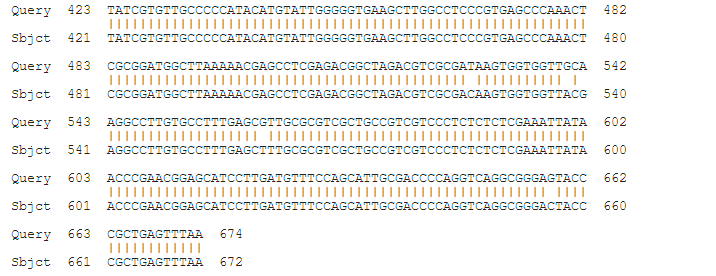


**H20** Alignment of the deduced amino acid sequences of gene of H1 (a) and GenBank (AB088597.1) (b).

**a.**

1 TTTCCGTAGG TGAACCTGCG GAAGGATCAT TGTCGAAGCC TAGAAAGCAT GACCGTGGAC

61 AAGTTGTTAA TCGTGGATGC ACTTGGATCC TTCGGGGTAC TCCATTTGCC TTCCACTCGG

121 TCGGTGATGA GCCTTGCGCC CATCGCTGAT TGAAACGAAT GAACCCCGGC GCGGATCGCG

181 CCAAGGAATC GAAACCGGAA GAGCTTGGCT CGGTGGCCCG TTCTCGGGCT GCCAAGGCCT

241 TAGGTCTATT CAAAATACCA TAACGACTCT CGGCAACGGA TATCTCGGCT CTCGCATCGA

301 TGAAGAACGT AGCAAAATGC GATACTTGGT GTGAATTGCA GAATCCCGTG AACCATCGAG

361 TTTTTGAACG CAAGTTGCGC CCGAAGCCAT TAGGCCGAGG GCACGTCTGC CTGGGCGTCA

421 CGTATCGTGT TGCCCCCATA CATGTATTGG GGGTGAAGCT TGGCCTCCCG TGAGCCCAAA

481 CTCGCGGATG GCTTAAAAAC GAGCCTCGAG ACGGCTAGAC GTCGCGATAA GTGGTGGTTG

541 CAAGGCCTTG TGCCTTTGAG CGTTGCGCGT CGCTGCCGTC TTCCCTCTCT CTCGAAATTA

601 TAACCCGAAC GGAGCATCCT TGATGTTTTC CAGCATTGCG ACCCCAGGTC AGGCGGGAGT

661 ACTCCGCTGA GTTTAA

**b.**


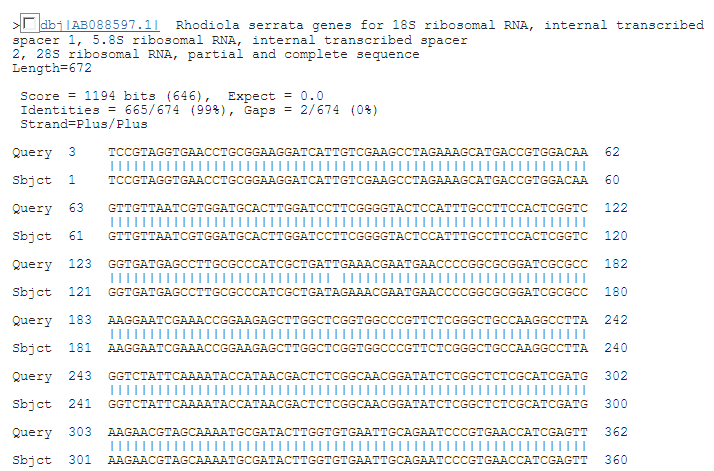


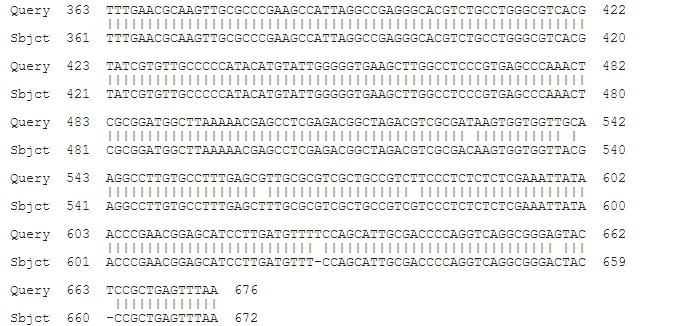


**H22** Alignment of the deduced amino acid sequences of gene of H1 (a) and GenBank (AB088597.1) (b).

**a.**

TTTCCGTAGG TGAACCTGCG GAAGGATCAT TGTCGAAGCC TAGAAAGCAT GACCGTGGAC AAGTTGTTAA TCGTGGATGC ACTTGGATCC TTCGGGGTAC TCCATTTGCC TTCCACTCGG TCGGTGATGA GCCTTGCGCC CATCGCTGAT TGAAACGAAT GAACCCCGGC GCGGATCGCG CCAAGGAATC GAAACCGGAA GAGCTTGGCT CGGTGGCCCG TTCTCGGGCT GCCAAGGCCT TAGGTCTATT CAAAATACCA TAACGACTCT CGGCAACGGA TATCTCGGCT CTCGCATCGA TGAAGAACGT AGCAAAATGC GATACTTGGT GTGAATTGCA GAATCCCGTG AACCATCGAG TTTTTGAACG CAAGTTGCGC CCGAAGCCAT TAGGCCGAGG GCACGTCTGC CTGGGCGTCA CGTATCGTGT TGCCCCCATA CATGTATTGG GGGTGAAGCT TGGCCTCCCG TGAGCCCAAA CTCGCGGATG GCTTAAAAAC GAGCCTCGAG ACGGCTAGAC GTCGCGATAA GTGGTGGTTG CAAGGCCTTG TGCCTTTGAG CGTTGCGCGT CGCTGCCGTC GTCCCTCTCT CTCGAAATTA TAACCCGAAC GGAGCATCCT TGATGTTTCC AGCATTGCGA CCCCAGGTCA GGCGGGAGTA CCCGCTGAGT TTAA

**b.**


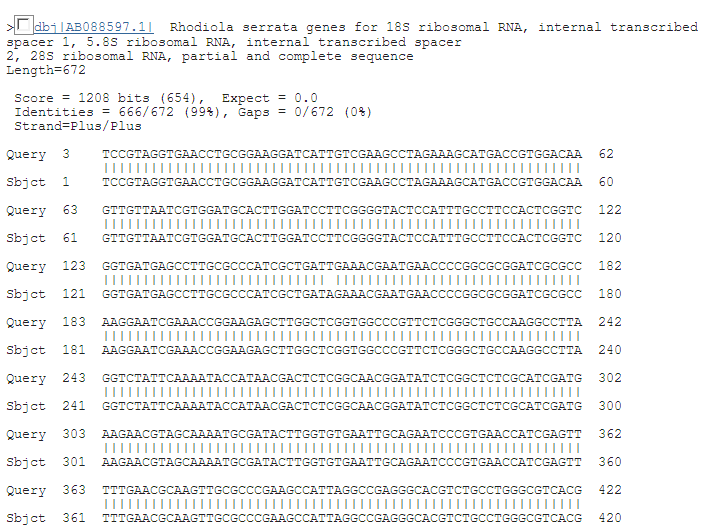


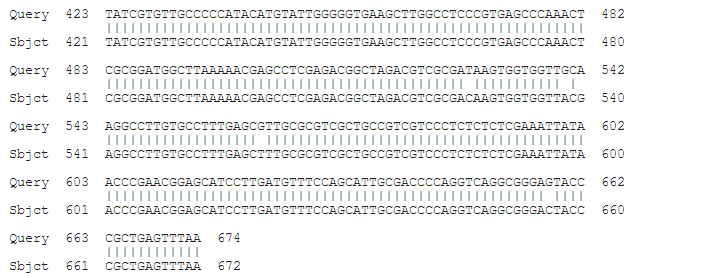


**H23** Alignment of the deduced amino acid sequences of gene of H1 (a) and GenBank (EU239665.1) (b).

**a.**

1 TTTCCGTAGG TGAACCTGCG GAAGGATCAT TGTCGAAGCC TAGAAAGCAT GACCGTGGAC

61 AAGTTGTTAA TCGTGGATGC ACTTGGATCC TTCGGGGTAC TCCATTTGCC TTCCACTCGG

121 TCGGTGATGA GCCTTGCGCC CATCGCTGAT TGAAACGAAT GAACCCCGGC GCGGATCGCG

181 CCAAGGAATC GAAACCGGAA GAGCTTGGCT CGGGGGCCCG TTCTCGGGCC GCCAAGGCCT

241 TAGGTCTATT CAAAATACCA TAACGACTCT CGGCAACGGA TATCTCGGCT CTCGCATCGA

301 TGAAGAACGT AGCAAAATGC GATACTTGGT GTGAATTGCA GAATCCCGTG AACCATCGAG

361 TTTTTGAACG CAAGTTGCGC CCGAAGCCAT TAGGCCGAGG GCACGTCTGC CTGGGCGTCA

421 CGTATCGTGT TGCCCCCATA CATGTATTGG GGGTGAAGCT TGGCCTCCCG TGAGCCCAAA

481 CTCGCGGATG GCTTAAAAAC GAGCCTCGAG ACGGTTAGAC GTCGCGACAA GTGGTGGTTA

541 CGAGGCCTTG CGCCTTTGAG CTTTGCGCGT CGCTGCCGTC GTCCCTCTCT CTCGAAATTA

601 TAACCCGAAC GGAGCATCCT CGATGTTTCC AGCATTGCGA CCCCAGGTCA GGCGGGAGTA

661 CCCGCTGAGT TTAA

**b.**


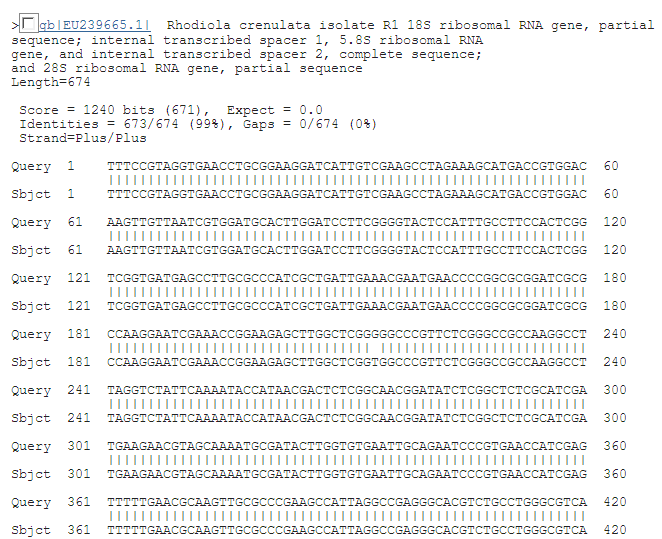

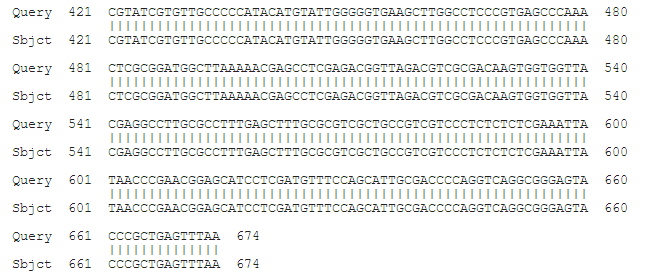


**H24** Alignment of the deduced amino acid sequences of gene of H1 (a) and GenBank (EU239666.1) (b).

**a.**

1 TTTCCGTAGG TGAACCTGCG GAAGGATCAT TGTCGAAGCC TAGAAAGCAT GACCGTGGAC

61 AAGTTGTTAA TCGTGGATGC ACTTGGATCC TTCGGGGTAC TCCATTTGCC TTCCACTCGG

121 TCGGTGATGA GCCTTGCGCC CATCGCTGAT TGAAACGAAT GAACCCCGGC GCGGATCGCG

181 CCAAGGAATC GAAACCGGAA GAGCTTGGCT CGGGGGCCCG TTCTCGGGCC GCCAAGGCCT

241 TAGGTCTATT CAAAATACCA TAACGACTCT CGGCAACGGA TATCTCGGCT CTCGCATCGA

301 TGAAGAACGT AGCAAAATGC GATACTTGGT GTGAATTGCA GAATCCCGTG AACCATCGAG

361 TTTTTGAACG CAAGTTGCGC CCGAAGCCAT TAGGCCGAGG GCACGTCTGC CTGGGCGTCA

421 CGTATCGTGT TGCCCCCATA CATGTATTGG GGGTGAAGCT TGGCCTCCCG TGAGCCCAAA

481 CTCGCGGATG GCTTAAAAAC GAGCCTCGAG ACGGTTAGAC GTCGCGACAA GTGGTGGTTA

541 CGAGGCCTTG CGCCTTTGAG CTTTGCGCGT CGCTGCCGTC GTCCCTCTCT CTCGAAATTA

601 TAACCCGAAC GGAGCATCCT CGATGTTTCC AGCATTGCGA CCCCAGGTCA GGCGGGAGTA

661 CCCGCTGAGT TTAA

**b.**


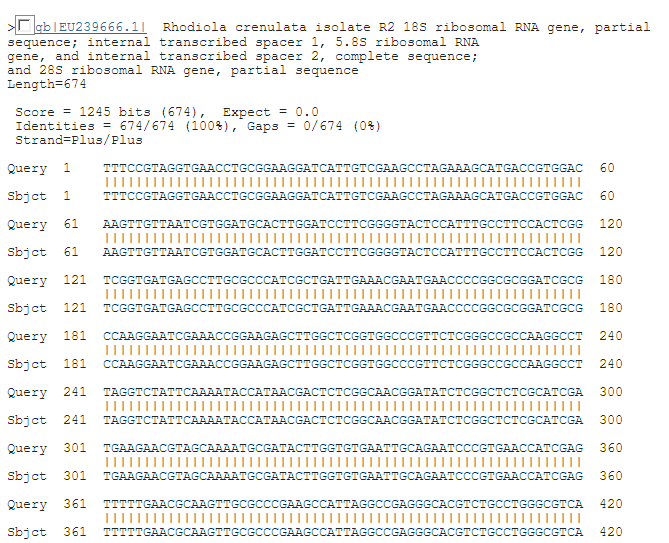


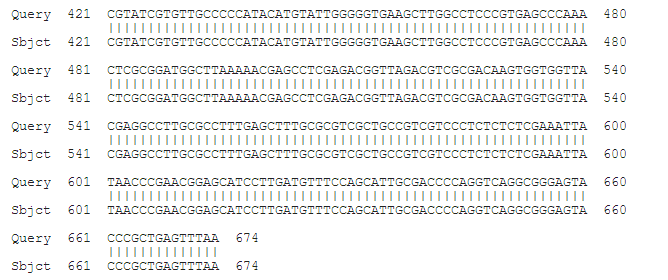


**H25** Alignment of the deduced amino acid sequences of gene of H1 (a) and GenBank (AB088597.1) (b).

**a.**

1 TTTCCGTAGG TGAACCTGCG GAAGGATCAT TGTCGAAGCC TAGAAAGCAT GACCGTGGAC

61 AAGTTGTTAA TCGTGGATGC ACTTGGATCC TTCGGGGTAC TCCATTTGCC TTCCACTCGG

121 TCGGTGATGA GCCTTGCGCC CATCGCTGAT TGAAACGAAT GAACCCCGGC GCGGATCGCG

181 CCAAGGAATC GAAACCGGAA GAGCTTGGCT CGGTGGCCCG TTCTCGGGCT GCCAAGGCCT

241 TAGGTCTATT CAAAATACCA TAACGACTCT CGGCAACGGA TATCTCGGCT CTCGCATCGA

301 TGAAGAACGT AGCAAAATGC GATACTTGGT GTGAATTGCA GAATCCCGTG AACCATCGAG

361 TTTTTGAACG CAAGTTGCGC CCGAAGCCAT TAGGCCGAGG GCACGTCTGC CTGGGCGTCA

421 CGTATCGTGT TGCCCCCATA CATGTATTGG GGGTGAAGCT TGGCCTCCCG TGAGCCCAAA

481 CTCGCGGATG GCTTAAAAAC GAGCCTCGAG ACGGCTAGAC GTCGCGATAA GTGGTGGTTG

541 CAAGGCCTTG TGCCTTTGAG CGTTGCGCGT CGCTGCCGTC GTCCCTCTCT CTCGAAATTA

601 TAACCCGAAC GGAGCATCCT TGATGTTCCA GCATTGCGAC CCCAGTCAGG CGGAGACACG

661 GTG

**b.**


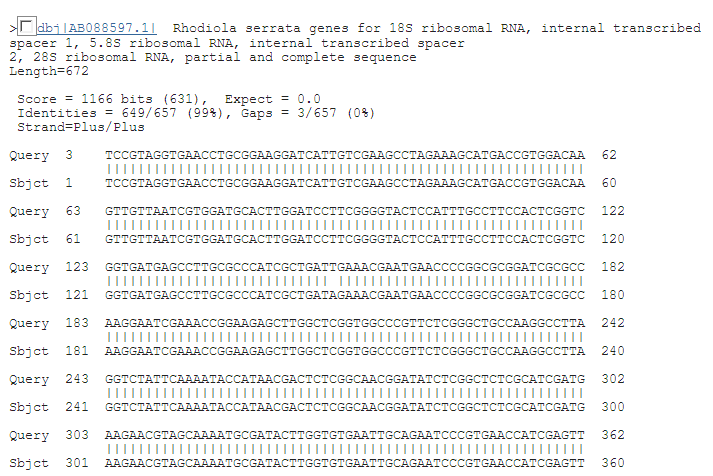


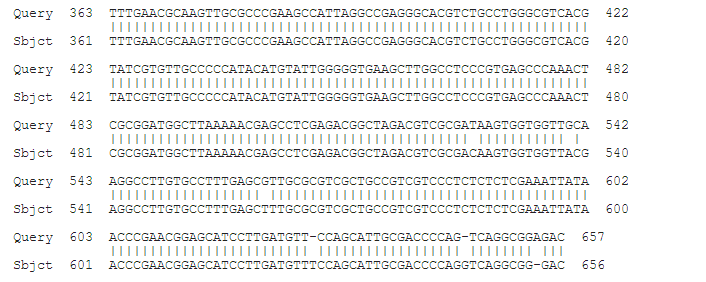


**H26** Alignment of the deduced amino acid sequences of gene of H1 (a) and GenBank (AY359900.1) (b).

**a.**

1 TCAGAAGTCG GGTACAAGGT TTCCGTAGGT GAACCTGCGG AAGGATCATT GTCGAAGCCT

61 AGAAAGCATG ACCGCGGACA AGTTGTTAAT CGTGGATGCA CTTGGATCCT TCGGGTACTC

121 CATTTGCCTT CCCCTCGGTC GGTGATGAGC CTTGCGCCCA TCGCTGATCG AAACGAATGA

181 ACCCCGGCGC GGATCGCGCC AAGGAATCGA AACCGAAAGA GCTTGGCTCG GTGGCCCGTT

241 CTCGGGGTGC CAGGGCCATA GGTCTATTCA AAATACCATA ACGACTCTCG GCAACGGATA

301 TCTCGGCTCT CGCATCGATG AAGAACGTAG CAAAATGCGA TACTTGGTGT GAATTGCAGA

361 ATCCCGTGAA CCATCGAGTT TTTGAACGCA AGTTGCGCCC GAAGCCATTA GGCCGAGGGC

421 ACGTCTGCCT GGGCGTCACG TATTGTGTTG CCCCCATACA TGTATTGGGT GTGAAGCTTG

481 GCCTCCCGTG AGCCCAAACT CGCGGATGGC TTAAAAACGA GCCTCGAGAC GGTTAGACGT

541 CGCGACAAGT GGTGGTTGCG AGGCCTTGTG CCTTTGAGCG TTGCGTGTCG CTGCCGTCGT

601 CCCTCTCTCT CGAAATTATA ACCCGAACGG AGCATCCTCG ATGTTTCCAG CATGCGACCC

661 CAGGTCAGGC GGTTGACCCG GA

**b.**


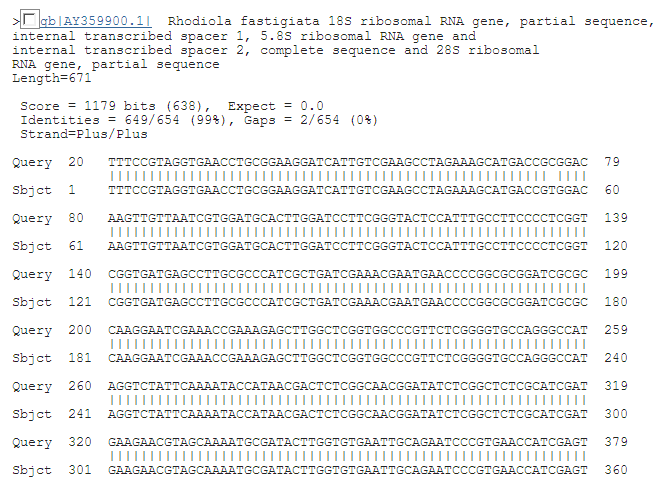


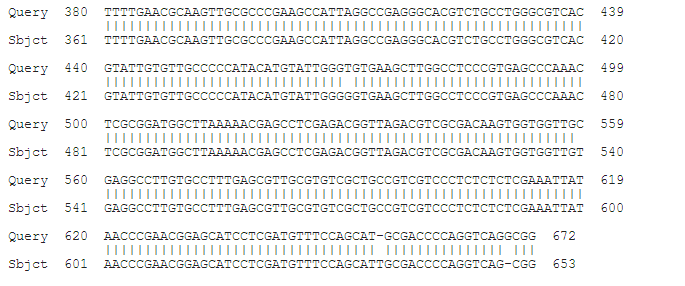


**H27** Alignment of the deduced amino acid sequences of gene of H1 (a) and GenBank (AB088597.1) (b).

**a.**

1 TTTCCGTAGG TGAACCTGCG GAAGGATCAT TGTCGAAGCC TAGAAAGCAT GACCGTGGAC

61 AAGTTGTTAA TCGTGGATGC ACTTGGATCC TTCGGGGTAC TCCATTTGCC TTCCACTCGG

121 TCGGTGATGA GCCTTGCGCC CATCGCTGAT TGAAACGAAT GAACCCCGGC GCGGATCGCG

181 CCAAGGAATC GAAACCGGAA GAGCTTGGCT CGGTGGCCCG TTCTCGGGCT GCCAAGGCCT

241 TAGGTCTATT CAAAATACCA TAACGACTCT CGGCAACGGA TATCTCGGCT CTCGCATCGA

301 TGAAGAACGT AGCAAAATGC GATACTTGGT GTGAATTGCA GAATCCCGTG AACCATCGAG

361 TTTTTGAACG CAAGTTGCGC CCGAAGCCAT TAGGCCGAGG GCACGTCTGC CTGGGCGTCA

421 CGTATCGTGT TGCCCCCATA CATGTATTGG GGGTGAAGCT TGGCCTCCCG TGAGCCCAAA

481 CTCGCGGATG GCTTAAAAAC GAGCCTCGAG ACGGCTAGAC GTCGCGATAA GTGGTGGTTG

541 CAAGGCCTTG TGCCTTTGAG CGTTGCGCGT CGCTGCCGTC GTCCCTCTCT CTCGAAATTA

601 TAACCCGAAC GGAGCATCCT TGATGTTTCC AGCATTGCGA CCCCAGGTCA GGCGGGAGTA

661 CCCGCTGAGT TTA

**b.**


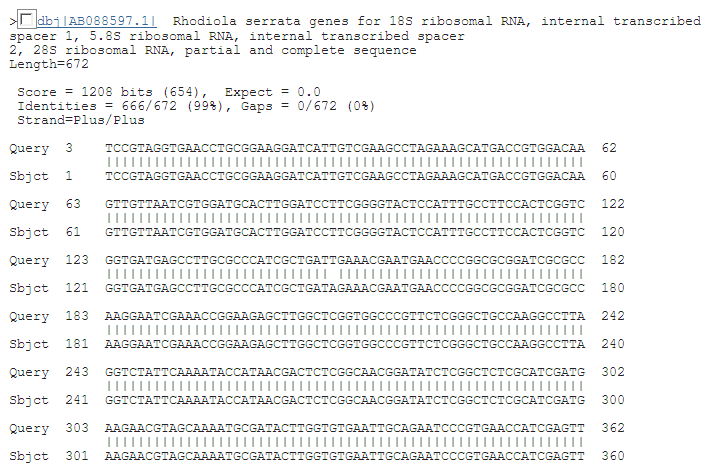


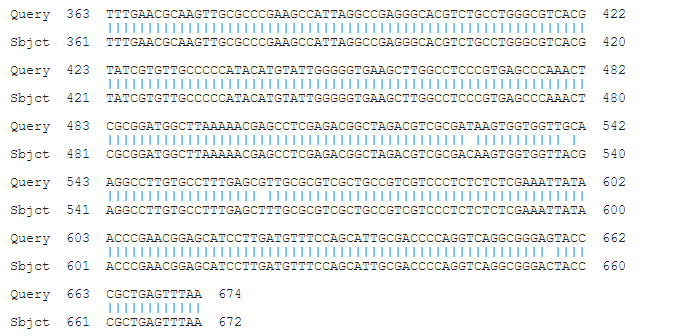


**H33** Alignment of the deduced amino acid sequences of gene of H1 (a) and GenBank (EU239669.1) (b).

**a.**

1 TTTCCGTAGG TGAACCTGCG GAAGGATCAT TGTCGAAGCC TAGAAAGCAT GACCGTGGAC

61 AAGTTGTTAA TCGTGGATGC ACTTGGATCC TTCGGGTACT CCATTTGCCT TCCACTCGGT

121 CGGTGATGAG CCTTGCGTCC ATTGCTGATC GAAACGAATG AACCCCGGCG CGGATCGCGC

181 CAAGGAATCA AAACCGAAAG AGCTTGGCTC GGTGGCCCGT TCTTGGGTTG CCAGGGCCAT

241 AGGTCTATTC AAAATACCAT AACGACTCTC GGCAACGGAT ATCTCGGCTC TCGCATCGAT

301 GAAGAACGTA GCAAAATGCG ATACTTGGTG TGAATTGCAG AATCCCGTGA ACCATCGAGT

361 TTTTGAACGC AAGTTGCGCC CGAAGCCATT AGGCTGAGGG CACGTCTGCC TGGGCGTCAC

421 ATATTGTGTT GCCCCCATAC ATGTATTGGG GGTGAAGCTT GGCCTCCCGT GAGCCCAAAC

481 TCGCGGATGG CTTAAAAACG AGCCTCGAGA CTGTTAGATG TCGCGATAAG TGGTGGTTAC

541 GAGGCCTTGT GCCTTTGAGC GTTGCGTGTC GCTGCCGTCG TCCCTCTCTC TCGAAATTAC

601 AACCCGAACG GAGCATCCTC GATGTTTCCA GCATTGCGAC CCCAGGTCAG GTGGGATCAC

661 CCGCTGAGTT TAA

**b.**


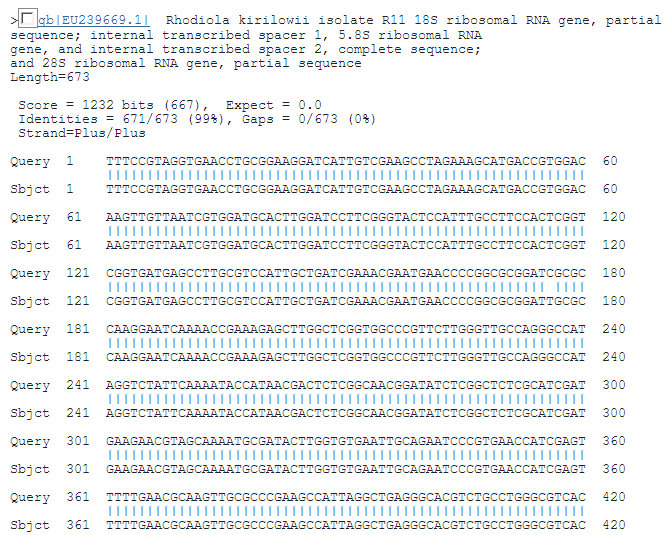

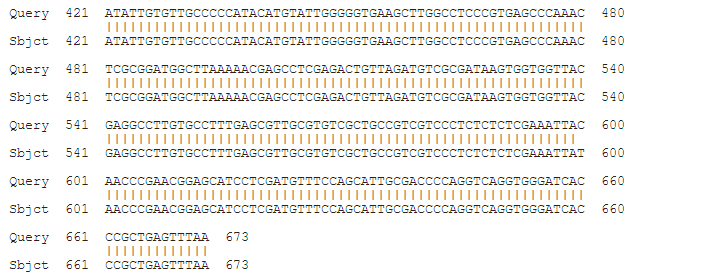


**H35** Alignment of the deduced amino acid sequences of gene of H1 (a) and GenBank (EU239669.1) (b).

**a.**

1 TTTCCGTAGG TGAACCTGCG GAAGGATCAT TGTCGAAGCC TAGAAAGCAT GACCGTGGAC

61 AAGTTGTTAA TCGTGGATGC ACTTGGATCC TTCGGGTACT CCATTTGCCT TCCACTCGGT

121 CGGTGATGAG CCTTGCGTCC ATTGCTGATC GAAACGAATG AACCCCGGCG CGGATCGCGC

181 CAAGGAATCA AAACCGAAAG AGCTTGGCTC GGTGGCCCGT TCTTGGGTTG CCAGGGCCAT

241 AGGTCTATTC AAAATACCAT AACGACTCTC GGCAACGGAT ATCTCGGCTC TCGCATCGAT

301 GAAGAACGTA GCAAAATGCG ATACTTGGTG TGAATTGCAG AATCCCGTGA ACCATCGAGT

361 TTTTGAACGC AAGTTGCGCC CGAAGCCATT AGGCTGAGGG CACGTCTGCC TGGGCGTCAC

421 ATATTGTGTT GCCCCCATAC ATGTATTGGG GGTGAAGCTT GGCCTCCCGT GAGCCCAAAC

481 TCGCGGATGG CTTAAAAACG AGCCTCGAGA CTGTTAGATG TCGCGATAAG TGGTGGTTAC

541 GAGGCCTTGT GCCTTTGAGC GTTGCGTGTC GCTGCCGTCG TCCCTCTCTC TCGAAATTAC

601 AACCCGAACG GAGCATCCTC GATGTTTCCA GCATTGCGAC CCCAGGTCAG GTGGGATCAC

661 CCGCTGAGTT TAA

**b.**


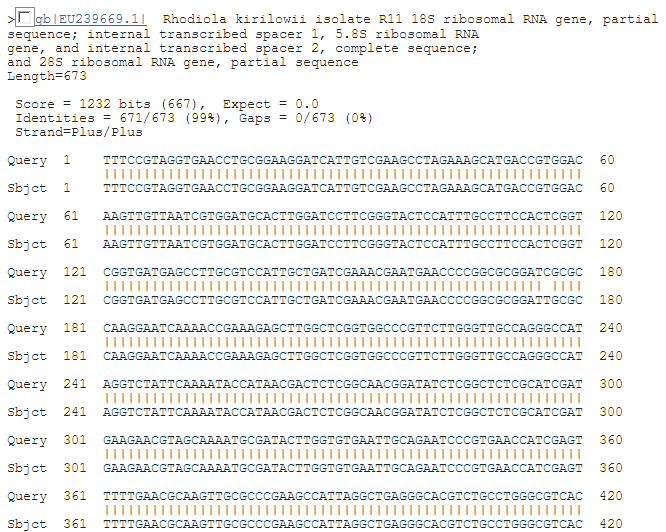

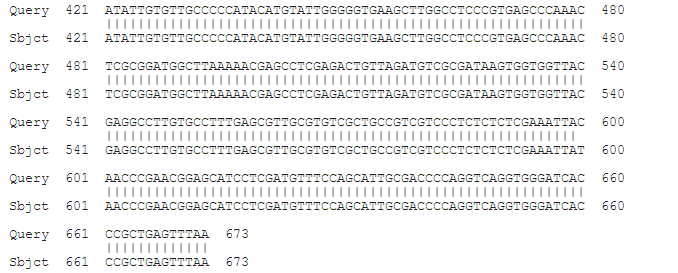


**H32** Alignment of the deduced amino acid sequences of gene of H1 (a) and GenBank (EU239666.1) (b).

**a.**

1 TTTCCGTAGG TGAACCTGCG GAAGGATCAT TGTCGAAGCC TAGAAAGCAT GACCGTGGAC

61 AAGTTGTTAA TCGTGGATGC ACTTGGATCC TTCGGGGTAC TCCATTTGCC TTCCACTCGG

121 TCGGTGATGA GCCTTGCGCC CATCGCTGAT TGAAACGAAT GAACCCCGGC GCGGATCGCG

181 CCAAGGAATC GAAACCGGAA GAGCTTGGCT CGGTGGCCCG TTCTCGGGCC GCCAAGGCCT

241 TAGGTCTATT CAAAATACCA TAACGACTCT CGGCAACGGA TATCTCGGCT CTCGCATCGA

301 TGAAGAACGT AGCAAAATGC GATACTTGGT GTGAATTGCA GAATCCCGTG AACCATCGAG

361 TTTTTGAACG CAAGTTGCGC CCGAAGCCAT TAGGCCGAGG GCACGTCTGC CTGGGCGTCA

421 CGTATCGTGT TGCCCCCATA CATGTATTGG GGGTGAAGCT TGGCCTCCCG TGAGCCCAAA

481 CTCGCGGATG GCTTAAAAAC GAGCCTCGAG ACGGTTAGAC GTCGCGACAA GTGGTGGTTA

541 CGAGGCCTTG CGCCTTTGAG CTTTGCGCGT CGCTGCCGTC GTCCCTCTCT CTCGAAATTA

601 TAACCCGAAC GGAGCATCCT TGATGTTTCC AGCATTGCGA CCCCAGGTCA GGCGGGAGTA

661 CCCGCTGAGT TTAA

**b.**


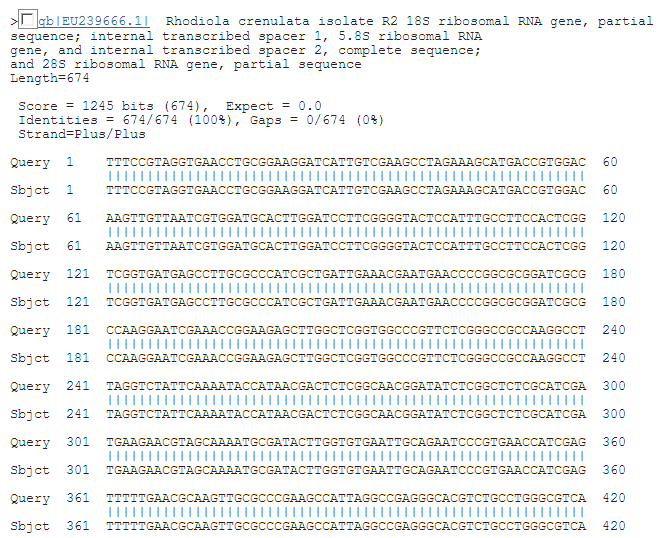


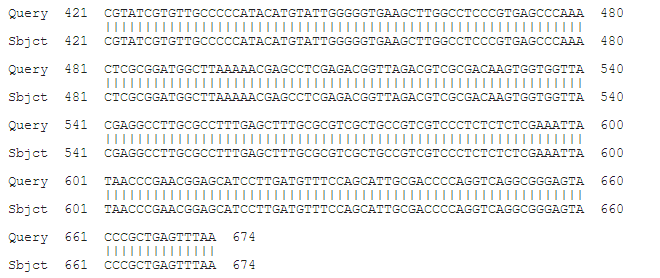


**Table 1S.** The 47 *Rhodiola* samples and the concentrations of the eight chemotaxonomic markers investigated in the samples.

| Name | Gallic acid | | Salidroside | | Tyrosol | | Catechin |  | Rhodionin | | Rosavin | | Rosarin | | Rosin | |
| --- | --- | --- | --- | --- | --- | --- | --- | --- | --- | --- | --- | --- | --- | --- | --- | --- |
|  | Content (%) | RSD (%) | Content (%) | RSD (%) | Content (%) | RSD (%) | Content (%) | RSD (%) | Content (%) | RSD (%) | Content (%) | RSD (%) | Content (%) | RSD (%) | Content (%) | RSD (%) |
| RC 1 | 0.277 | 1.98 | 0.787 | 0.93 | 0.269 | 1.34 | ND | - | 0.156 | 2.04 | ND | - | ND | - | ND | - |
| RC 2 | 0.419 | 0.23 | 0.216 | 0.79 | 0.905 | 1.25 | ND | - | 0.181 | 2.89 | ND | - | ND | - | ND | - |
| RC 3 | 0.441 | 0.45 | 0.197 | 1.29 | 0.930 | 0.63 | ND | - | 0.033 | 2.66 | ND | - | ND | - | ND | - |
| RC 4 | 0.445 | 0.67 | 1.568 | 0.62 | 0.983 | 0.69 | ND | - | 0.232 | 2.12 | ND | - | ND | - | ND | - |
| RC 5 | 0.611 | 0.33 | 0.921 | 0.39 | 1.666 | 0.41 | 0.134 | 1.22 | 0.191 | 1.04 | ND | - | ND | - | ND | - |
| RC 6 | 0.403 | 0.56 | 0.895 | 0.81 | 0.608 | 1.98 | ND | - | 0.156 | 1.50 | ND | - | ND | - | ND | - |
| RC 7 | 0.678 | 1.23 | 0.495 | 0.69 | 0.701 | 1.37 | ND | - | 0.099 | 2.93 | ND | - | ND | - | ND | - |
| RC 8 | 0.510 | 0.45 | 0.781 | 1.39 | 0.669 | 1.01 | ND | - | 0.172 | 1.34 | ND | - | ND | - | ND | - |
| RC 9 | 0.432 | 0.98 | 0.622 | 1.81 | 0.500 | 2.07 | ND | - | 0.259 | 0.89 | ND | - | ND | - | ND | - |
| RC 10 | 0.152 | 1.76 | 1.690 | 1.91 | 0.119 | 2.89 | ND | - | 0.287 | 1.02 | ND | - | ND | - | ND | - |
| RC 11 | 0.441 | 0.56 | 0.793 | 1.22 | ND | - | ND | - | ND | - | ND | - | ND | - | ND | - |
| RC 12 | 0.105 | 2.10 | 0.562 | 0.47 | ND | - | ND | - | ND | - | ND | - | ND | - | ND | - |
| RC 13 | 0.808 | 0.34 | 1.009 | 0.39 | 0.677 | 2.98 | ND | - | 0.765 | 0.34 | ND | - | ND | - | ND | - |
| RC 14 | 0.141 | 1.56 | 0.065 | 2.73 | 0.228 | 2.10 | ND | - | 0.016 | 2.66 | ND | - | ND | - | ND | - |
| RC 15 | 0.400 | 0.91 | 1.002 | 0.94 | 0.526 | 0.89 | ND | - | 0.196 | 0.39 | ND | - | ND | - | ND | - |
| RC 16 | 0.788 | 0.71 | 1.665 | 0.29 | 0.696 | 0.37 | ND | - | 0.570 | 1.90 | ND | - | 0.157 |  | ND | - |
| RC 17 | 0.39 | 1.29 | 0.518 | 0.71 | 0.813 | 1.25 | 0.414 | 0.36 | 0.075 | 2.67 | ND | - | ND | - | ND | - |
| RC 18 | 0.405 | 1.85 | 0.683 | 0.39 | 0.765 | 1.49 | 0.125 | 1.49 | 0.206 | 1.04 | ND | - | ND | - | ND | - |
| RC 19 | 0.155 | 1.69 | 1.499 | 1.23 | 0.595 | 2.05 | ND | - | 0.104 | 1.49 | ND | - | ND | - | ND | - |
| RS20 | 0.472 | 1.92 | 0.535 | 0.39 | 0.036 | 1.04 | ND | - | 0.088 | 2.04 | ND | - | 0.130 | 0.88 | 0.140 | 1.37 |
| RS21 | 0.578 | 2.03 | 0.960 | 0.89 | 0.204 | 2.79 | ND | - | 0.033 | 2.49 | ND | - | 0.324 | 1.27 | 0.276 | 0.89 |
| RH22 | 0.154 | 1.49 | ND | - | ND | - | 0.063 | 2.84 | ND | - | 0.579 | 1.11 | 0.234 | 1.94 | 0.683 | 0.33 |
| RSE23 | 0.309 | 0.49 | 0.622 | 0.88 | 0.008 | 2.88 | ND | - | 0.009 | 2.88 | 0.032 | 0.67 | ND | - | ND | - |
| RSE 24 | 0.143 | 1.65 | 0.049 | 2.89 | 0.017 | 2.49 | ND | - | 0.010 | 2.19 | 0.057 | 1.98 | ND | - | ND | - |
| RSE 25 | 0.258 | 1.36 | 0.025 | 2.78 | 0.013 | 2.37 | ND | - | 0.008 | 2.79 | 0.012 | 2.38 | ND | - | ND | - |
| RSE 26 | 0.280 | 1.67 | 0.172 | 0.89 | 0.047 | 2.48 | ND | - | 0.024 | 2.88 | 0.009 | 2.45 | ND | - | ND | - |
| RSE 27 | 0.407 | 0.29 | 0.646 | 0.59 | ND | - | ND | - | 0.016 | 2.19 | 0.005 | 2.91 | ND | - | ND | - |
| RSE 28 | 0.180 | 1.08 | ND | - | 0.012 | 2.01 | ND | - | ND | - | 0.002 | 2.68 | ND | - | ND | - |
| RSE 29 | 0.456 | 1.92 | 0.172 | 0.94 | 0.005 | 2.59 | ND | - | 0.011 | 2.61 | 0.045 | 2.01 | ND | - | ND | - |
| RSE 30 | 0.301 | 0.83 | 0.017 | 1.79 | 0.003 | 2.15 | ND | - | 0.019 | 2.74 | 0.078 | 2.92 | ND | - | ND | - |
| RSE 31 | 0.239 | 1.28 | 0.018 | 1.98 | 0.013 | 2.83 | ND | - | 0.013 | 2.78 | 0.011 | 1.78 | ND | - | 0.001 | 2.94 |
| RSE 32 | 0.323 | 0.79 | 0.013 | 2.19 | 0.015 | 2.31 | ND | - | 0.016 | 2.19 | 0.001 | 2.58 | ND | - | ND | - |
| RSE 33 | 0.328 | 0.30 | 0.017 | 2.34 | 0.015 | 2.91 | ND | - | 0.008 | 2.77 | 0.007 | 2.75 | ND | - | ND | - |
| RSE 34 | 0.296 | 0.83 | ND | - | ND | - | ND | - | ND | - | 0.009 | 1.39 | ND | - | ND | - |
| RSE 35 | 0.324 | 0.59 | ND | - | ND | - | ND | - | ND | - | 0.098 | 1.78 | ND | - | ND | - |
| RSE 36 | 0.193 | 1.98 | ND | - | ND | - | ND | - | ND | - | 0.071 | 1.93 | ND | - | ND | - |
| RSE 37 | 0.277 | 0.56 | ND | - | ND | - | ND | - | 0.024 | 2.11 | 0.098 | 1.77 | ND | - | ND | - |
| RF38 | 0.192 | 0.86 | 1.179 | 0.39 | 0.451 | 1.78 | 0.028 | 2.76 | 0.005 | 1.39 | ND | - | ND | - | ND | - |
| RR39 | 0.145 | 1.98 | 1.097 | 0.04 | 0.569 | 2.03 | ND | - | 0.154 | 1.98 | 0.536 | 1.28 | 0.110 | 2.10 | 0.090 | 2.67 |
| RK 40 | 0.369 | 0.33 | ND | - | ND | - | ND | - | 0.043 | 1.26 | ND | - | 0.126 | 1.47 | ND | - |
| RK 41 | 0.289 | 0.38 | 0.174 | 1.79 | 0.170 | 2.34 | ND | - | ND | - | ND | - | ND | - | ND | - |
| RK 42 | 0.397 | 0.69 | 0.373 | 0.29 | 0.380 | 2.18 | ND | - | ND | - | ND | - | ND | - | ND | - |
| RK 43 | 0.143 | 1.88 | 0.326 | 0.62 | 0.888 | 1.34 | ND | - | ND | - | ND | - | ND | - | ND | - |
| RK 44 | 1.089 | 0.63 | 0.806 | 0.74 | 0.149 | 1.73 | ND | - | ND | - | ND | - | ND | - | ND | - |
| RK 45 | 0.357 | 1.38 | 0.536 | 0.91 | 0.032 | 2.87 | ND | - | ND | - | ND | - | ND | - | ND | - |
| RK 46 | 0.257 | 0.36 | 0.503 | 0.28 | 0.029 | 2.94 | ND | - | ND | - | ND | - | ND | - | ND | - |
| RK 47 | 0.366 | 0.98 | 0.504 | 0.69 | 0.070 | 2.10 | ND | - | ND | - | ND | - | ND | - | ND | - |

ND = not detectable

1. ^*^ *Corresponding author*:

   Prof. Aiping Lu, Institute of Basic Research in Clinical Medicine, China Academy of Chinese Medical Sciences, Beijing 100700, China. Tel.: +86 10 64067611, Fax: +86 10 64013896.

   *E-mail address*: [lap64067611@126.com](mailto:catcm@public.bta.net.cn) (A.P. Lu).

   ^§^ These authors contributed equally to this work. [↑](#footnote-ref-2)
